# Detection of *pks* Island mRNAs Using Toehold Sensors in *Escherichia coli*

**DOI:** 10.3390/life11111280

**Published:** 2021-11-22

**Authors:** Taeyang Heo, Hansol Kang, Seungdo Choi, Jongmin Kim

**Affiliations:** Department of Life Sciences, Pohang University of Science and Technology, Pohang 37673, Korea; hty1998@postech.ac.kr (T.H.); hskang0405@postech.ac.kr (H.K.); choisd@postech.ac.kr (S.C.)

**Keywords:** RNA synthetic biology, toehold switch, pathogenicity island, *pks* island, molecular diagnostics

## Abstract

Synthetic biologists have applied biomolecular engineering approaches toward the goal of novel biological devices and have shown progress in diverse areas of medicine and biotechnology. Especially promising is the application of synthetic biological devices towards a novel class of molecular diagnostics. As an example, a de-novo-designed riboregulator called toehold switch, with its programmability and compatibility with field-deployable devices showed promising in vitro applications for viral RNA detection such as Zika and Corona viruses. However, the in vivo application of high-performance RNA sensors remains challenging due to the secondary structure of long mRNA species. Here, we introduced ‘Helper RNAs’ that can enhance the functionality of toehold switch sensors by mitigating the effect of secondary structures around a target site. By employing the helper RNAs, previously reported *mCherry* mRNA sensor showed improved fold-changes in vivo. To further generalize the Helper RNA approaches, we employed automatic design pipeline for toehold sensors that target the essential genes within the *pks* island, an important target of biomedical research in connection with colorectal cancer. The toehold switch sensors showed fold-changes upon the expression of full-length mRNAs that apparently depended sensitively on the identity of the gene as well as the predicted local structure within the target region of the mRNA. Still, the helper RNAs could improve the performance of toehold switch sensors in many instances, with up to 10-fold improvement over no helper cases. These results suggest that the helper RNA approaches can further assist the design of functional RNA devices in vivo with the aid of the streamlined automatic design software developed here. Further, our solutions for screening and stabilizing single-stranded region of mRNA may find use in other in vivo mRNA-sensing applications such as cas13 crRNA design, transcriptome engineering, and trans-cleaving ribozymes.

## 1. Introduction

Synthetic biology is a burgeoning field that aims to design novel biological components, networks, and organisms by combining biological knowledge and technology with engineering principles [1,2]. Over the past decades, continued progress in the ability to redesign biological systems has succeeded in the construction of synthetic biological devices such as toggle switches [3,4], oscillators [4,5], counters [6], memory systems [7], pulse generators [8,9], and majority sensors [10]. The growing repertoire of sophisticated genetic circuitry in synthetic biological systems could find applications in medical and industrial fields, paving the way for precision medicine [11], cancer therapy [12,13], vaccine developments [14], and biosensors [15]. Despite numerous successful developments, the underlying limitations of natural and engineered biological circuit components, such as undefined compatibility, low dynamic range, poor predictability, and crosstalk, make it challenging to realize the next level of sophisticated synthetic biological designs that will drive future innovations. 

**Figure 1:**
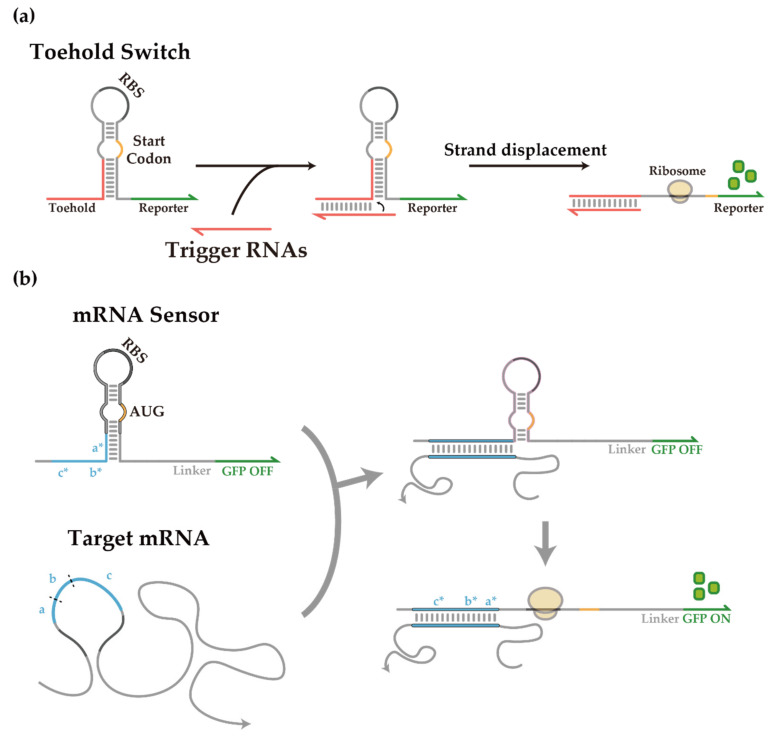
De-novo-designed toehold switch and toehold switch-based mRNA sensor. (**a**) Scheme of toehold switch operation. Toehold switches repress translation through a programmed hairpin structure sequestering the RBS and the start codon. RNA-RNA interaction upon the introduction of trigger RNAs completes a branch migration process with the switch hairpin to expose the RBS and start codon, thereby initiating translation of the downstream gene. (**b**) Scheme of toehold switch-based mRNA sensors. Domains a, b, and c indicate the target site within an mRNA, and domains a*, b*, and c* within the mRNA sensor are the reverse complementary sequences to each domain.

RNA-based synthetic gene regulatory components have an advantage that RNA-RNA interaction can be predicted via Watson–Crick base pairing. Therefore, synthetic biologists have endeavored to devise a novel riboregulator that controls transcription and/or translation in response to cognate RNAs [16,17,18]. For instance, toehold switches are de-novo-designed riboregulators that regulate translation initiation of a downstream gene by sequestering the ribosome binding site (RBS) and starting codon [19] (Figure 1a). The toehold switch design is mostly free from sequence constraints compared to earlier synthetic riboregulators [20], and consequently, could achieve a wide dynamic range and high programmability [19]. Several recent works employed toehold switches for synthetic biological circuitry, including cellular logic computation [21], translational repressing riboregulators [22], incoherent feed-forward loop circuits [9], synthetic transcription terminators [23], protein quality control system [24], and modulators of riboswitch circuits [25]. 

The versatility of toehold switches can be further showcased by recent developments of paper-based toehold switch systems as in vitro RNA detection platforms for Zika virus detection [26], Coronavirus detection [27], and gut microbiota analysis [28] in combination with well-known isothermal RNA amplification techniques (e.g., nucleic acid sequence-based amplification (NASBA) [29,30] or reverse transcription loop-mediated isothermal amplification (RT-LAMP) [31]). The flexibility of toehold switches may find use in combination with the CRISPR-Cas system for in vivo genome editing [32,33] and in situ gut microbiome engineering [34,35,36]. For instance, the translation control of toehold switches can limit the expression of Cas effectors to reduce cytotoxicity and off-target effects [37,38,39,40], and designed RNA self-assembly can enhance the complexity of signal integration in vivo beyond the current practice [41,42,43]. Since toehold switches have less cell burden and shorter response time compared to protein regulators [19,44,45], these approaches may result in a novel suite of synthetic RNA components for in vivo applications that integrates multiple cellular RNA signals. In principle, toehold switch sensors can target arbitrary RNA sequences as shown previously for *mCherry* mRNA and antibiotic resistance gene transcripts (Figure 1b) [19]. Still, detecting long mRNA species using toehold switch sensors typically shows reduced sensitivity [22,46], possibly due to a strong secondary structure within mRNA targets [19]. Even though RNA destabilizing chaperons have been studied for the unwinding of misfolded RNAs [47], they were not generally applicable to an arbitrary RNA. Previous works also have shown that helper oligonucleotide can be applied for disruption of RNA secondary structure by heat denaturation in vitro [48] or in situ [48,49], yet the applicability of the same strategy in vivo remains unclear. Therefore, novel developments to enhance toehold switch functionality in its native context remain an unmet need.

Pathogenicity islands (PAIs) are a group of virulent genomic islands which can be transmitted through horizontal gene transfer [50]. The *pks* island, one of the most well-known PAIs, mainly found in *Escherichia coli* belongs to phylogroup B2 [51,52] and produces genotoxin called colibactin [53]. Colibactin induces DNA double-strand breaks in epithelial cells [53] and has been linked to colorectal cancer [54,55,56] and inflammatory bowel diseases [57,58]. Interestingly, the *pks* island is also present in *E. coli* Nissle 1917, a probiotic strain widely used in clinical studies [59,60,61,62,63]. In particular, it is surprising that *pks* island harboring *E. coli* can demonstrate probiotic activity [64,65], antibiotic activity [66], or analgesic lipopeptide production [67]. Therefore, repurposing toehold switch sensors to target genes within the *pks* island can open up new possibilities to determine the activity of these genetic elements and potentially regulate their activities given appropriate molecular inputs. 

Here, we investigated the ‘Helper-assisted mRNA sensing (HAM)’ system that enhances output signals of toehold switch sensors with an automated design algorithm to find accessible target sequences within long mRNA molecules. The helper RNAs alleviate local secondary structures by binding to the upstream and/or downstream of the target sequence within mRNA molecule to unwind local structures formed via interaction of target sequence with its immediate neighboring domains. The previously reported *mCherry* mRNA sensor showed improved fold changes after introducing helper RNAs while maintaining mCherry expression levels. Next, we applied the HAM system to automatically designed *pks* island mRNA sensors. While the detection of full-length mRNAs by toehold switch sensors showed relatively low output fold-changes compared to synthetic short triggers, the introduction of the HAM system partially restored output signals with up to 10 fold increases compared to the absence of helper RNAs. In summary, our solutions for reducing local structures via HAM system at the molecular level and screening for structure-free target sites within a long mRNA molecule via an automated design algorithm helped identify highly functional toehold switch sensors for the *pks* island mRNAs. 

## 2. Materials and Methods

### 2.1. Plasmid Construction and E. coli Strains

The following *E. coli* strains were used in this study: BL21 AI (F^−^ *omp*T *hsd*S_B_ (r_B_^−^ m_B_^−^) *gal dcm ara*B::T7RNAP-*tet*A) and DH5α (*endA1 recA1 gyrA96 thi-1 glnV44 relA1 hsdR17*(r_K_^−^ m_K_^+^) λ^−^).

Backbones for the plasmids used in this research were taken from the commercial vectors pET15b, pCDFDuet, or pCOLADuet (EMD Millipore). All the target RNAs and noncognate decoys of toehold switch sensors were constructed in pET15b. Toehold switch sensors were constructed in pCOLADuet. Helper RNAs were constructed in pCDFDuet. All constructs were cloned via Gibson Assembly [68], circular polymerase extension cloning (CPEC) [69], and/or round-the-horn site-directed mutagenesis [70]. Plasmid architecture and specific part sequences are listed in Tables S1–S9. Plasmids were constructed in *E. coli* DH5α and purified using the EZ-PureTM plasmid Prep Kit. Ver. 2 (Enzynomics). Plasmid sequences were confirmed by DNA sequencing after every cloning step. Plasmids were transformed through chemical transformation.

### 2.2. Cell Culture and Induction Condition

For in vivo experiments, *E. coli* BL21 AI strain was used, which contains chromosomally integrated T7 RNA polymerase under the control of arabinose-inducible P_BAD_ promoter. For in vivo experiments, chemically transformed *E. coli* BL21 AI cells were cultured on LB agar plates (1.5% agar) with appropriate antibiotics: pCOLADuet (50 μg/mL Kanamycin), pCDFDuet (50 μg/mL Spectinomycin), pET15b (100 μg/mL Ampicillin). Single colonies were inoculated into 1 mL LB liquid medium with appropriate antibiotics. These cells were grown overnight (~16 h) in 96-well plates with shaking at 800 rpm and 37 °C. Overnight cultures were diluted 1/100-fold into fresh medium and returned to shaking (800 rpm, 37 °C). After 80 min, cell cultures were induced with 0.2% arabinose and returned to the shaker (800 rpm, 37 °C) until fluorescence measurement after 3 h and 30 min. 

### 2.3. Fluorescence Measurements Using Flow Cytometry

GFP fluorescence was measured using flow cytometry (CytoFLEX S, Beckman Coulter, Brea, CA, USA) after fixation at Microbiome Core Research Support Center of Korea Basic Science Institute (KBSI). The cell pellet was resuspended with 2% (*w*/*v*) para-formaldehyde solution and fixed for 15 min at room temperature. After fixation, samples were washed twice using 1× phosphate-buffered saline (PBS). Fixed cells were diluted by a factor of ~5 into 1× PBS. Cells were detected using a forward scatter (FSC) trigger, and at least 100,000 events were recorded for each measurement. The cell population was gated according to the FSC and side scatter (SSC) distributions as described previously [19]. To evaluate circuit output, the fluorescence of GFPmut3b-ASV was measured on a FITC channel, excited with a 488-nm, and detected with a 525/40-nm bandpass filter. GFP fluorescence histograms yielded unimodal population distributions, and the geometric mean was employed for the average fluorescence across the approximately log-normal fluorescence distribution from three biological replicates. GFP ON/OFF fold changes were then calculated by taking the average GFP fluorescence from the cognate RNA expressing case and dividing it by the GFP fluorescence from the noncognate RNA expressing case. Cellular autofluorescence was subtracted before determining ON/OFF ratios. *p*-values are calculated through the Student’s *t*-test. 

### 2.4. Quantitative Reverse-Transcription PCR

Cell culture and induction were performed in the same manner as the flow cytometry analysis. In DNase/RNase-free condition, total RNA was extracted using RiboEx (GeneAll, Seoul, Korea) reagent. Sample concentration and purity were measured using a BioTek Synergy H1 plate reader (BioTek, Winooski, VT, USA). cDNA was synthesized with 1 μg of total RNA as the template using random primers for whole-cell RNA reverse transcription (RT). The concentration of synthesized cDNAs was measured using the plate reader, then 1 μg of cDNA was diluted 1/40-fold. The cDNA was then used for subsequent analysis in a quantitative PCR step in Stratagene Mx3000P (Agilent Technologies, Santa Clara, CA, USA) with the following conditions: 50 °C for 2 min, 95 °C for 10 min, followed by 40 cycles of 95 °C for 15 s and 60 °C for 1 min. The number of replicates was three for each condition. All measurements were followed by melting curve analysis. Ct values were analyzed using MxPRO software (Agilent Technologies, Santa Clara, CA, USA). Primer sequences used in the article are listed in Supplementary Information (Table S10). 

### 2.5. In Silico Toehold Switch Sensor Design for clb ORFs

For in silico design of toehold switch sensors, NUPACK 4.0.0.25 [71,72,73,74,75] was used. In this paper, all toehold switch sensor designs followed the design of *mCherry* mRNA sensors introduced in the previous work [19]. The sequence of ORFs in *pks* island was obtained from previous reports [76]. By using NUPACK, we calculated the regional minimum free energy (MFE) structure of ORFs with a window of 400 nt and evaluated the accessibility within the central 200 nt. Both terminal ends of 100 nts within the 400 nt window were excluded due to the potential low reliability of predicted structures. However, when scoring the 5′ end or 3′ end of the mRNA, the exclusion of terminal ends was not performed. This in silico screening process was repeated for every 30 nt intervals, the size of a cognate trigger, until reaching the 3′ end, to completely tile the full length of mRNAs. Toehold switch sensors were automatically designed for all possible target regions, and those that contained in-frame stop codons were subsequently removed. Two highly accessible regions and two highly structured regions, and another region, were selected for experimental validation. Since NUPACK 4.0.0.25 is available in Python 3.8 [77], this toehold switch sensor design process is entirely automated. For data sorting and processing, pandas [78], an open library, was used. 

## 3. Results

### 3.1. Helper-Assisted mRNA Sensing of Toehold Switch

**Figure 2:**
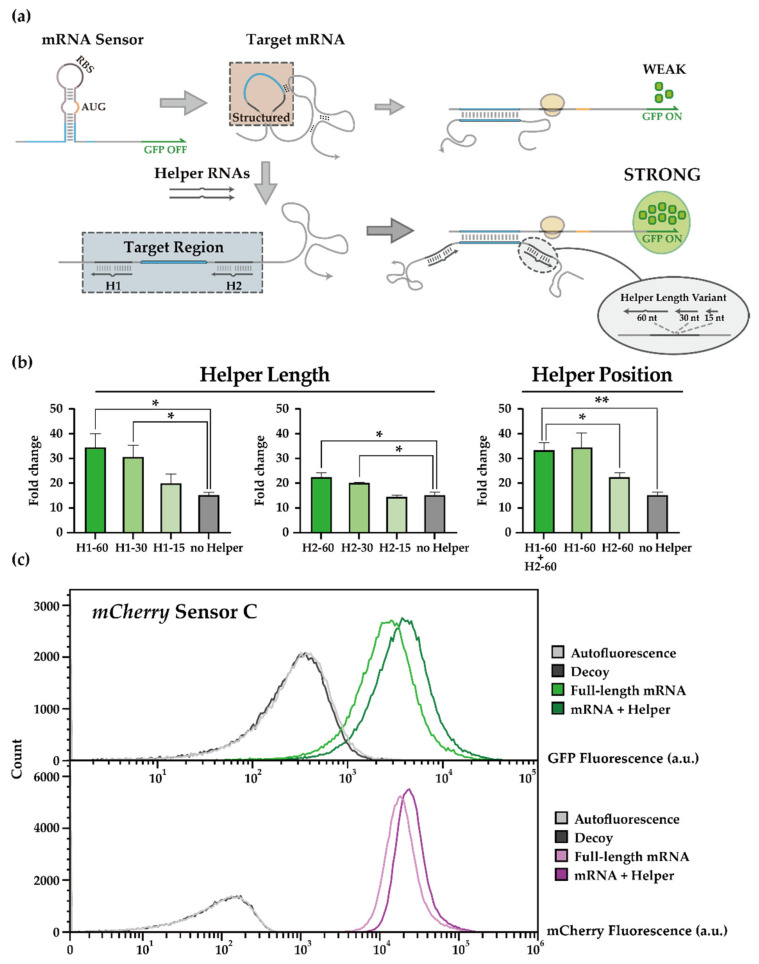
Helper-assisted mRNA sensing (HAM) platform. (**a**) Scheme of the helper-assisted mRNA sensor activation. Interaction between an mRNA sensor and a target mRNA can be hampered by a strong secondary structure within and around the target region of mRNA. Helper RNAs can bind to the upstream and/or downstream of the target region of the mRNA sensor such that the target region becomes relatively free of secondary structure and can interact with toehold switch sensors favorably. (**b**) In vivo characterization of helper RNAs for *mCherry* mRNA sensor. GFP fold changes for different sets of helper RNAs are listed by length (Left) or position (Right). H1 indicates helper RNA strands that bind to the upstream of a target site, while H2 indicates helper RNAs for the downstream domain with the numbers to indicate the length of helper RNAs (60, 30, and 15 nt). Fold changes were calculated by dividing the GFP fluorescence in the ON state by the GFP fluorescence in its OFF state. GFP fluorescence measurements were performed on flow cytometry. (two-tailed Student’s *t*-test; * *p* < 0.05; ** *p* < 0.01; Error bars indicate ± SEM). Cellular autofluorescence was subtracted before determining ON/OFF ratios. (**c**) Flow cytometry GFP and mCherry fluorescence histograms for a previously reported *mCherry* sensor with or without helper RNA in the presence of cognate full-length trigger RNA or decoy RNA. Autofluorescence level was measured from cells not bearing a GFP-expressing plasmid.

Toehold switch sensors for detecting *mCherry* mRNA were previously characterized [19], where the presence of *mCherry* mRNA inputs leads to the unwinding of hairpin structure around the RBS and start codon such that the downstream GFP output can be translated. Despite successful demonstration, toehold mRNA sensors produced relatively low ON levels compared to those developed for short synthetic triggers, possibly due to the secondary structures present in long mRNA input molecules [19,46]. To overcome this limitation, we decided to test “Helper RNAs” that help unravel RNA secondary structures around the target site (Figure 2a), thereby increasing the accessibility of the target sequence. An analogous strategy was shown to be effective in vitro and in situ [49,79], but the applicability of this strategy in vivo has not been verified. 

We set out to test several such helper RNA variants to explore the impact of RNA lengths and target domains. Specifically, helper RNAs of 15 nt, 30 nt, and 60 nt were employed that targeted upstream and/or downstream of the target site of previously reported *mCherry* mRNA sensors [19]. To avoid physical interference, the helper RNAs targeted domains separated from the domains targeted by toehold switch sensors by 3-nt-gap. Performance of helper RNAs was evaluated in the *E. coli* BL21 AI strain, where genomically encoded T7 RNA polymerase was induced by arabinose. Unless otherwise noted, the same conditions were employed for other experiments herein (see Methods). The fold change of GFP outputs for a given *mCherry* sensor was enhanced by introducing helper RNAs (Figure 2b, left), where helper RNAs of 30 nt or longer were particularly effective. Moreover, the helper RNAs targeting upstream of the original target domain seemed more effective (Figure 2b, right) with improvements up to 34.49 or 22.42 folds for upstream or downstream helpers, respectively. For 60-nt helper RNAs, a 3-nt-bulge in the middle of 60 nt helper RNA was introduced to reduce RNase activity that might disrupt helper-mRNA interactions [80]. Introducing helper RNAs upstream and downstream around the target site showed small improvements over helper RNAs for upstream cases. In previous works [49], stretching both sides adjacent to the target site could help sense the target RNA in vitro. Therefore, to balance the design efforts and potential benefits, we decided to use a pair of 30 nt helper RNAs in the next stage of further investigations. 

In conclusion, we observed that the output of the *mCherry* mRNA sensor was further enhanced with the introduction of helper RNAs (Figure 2c). However, another *mCherry* mRNA sensor with good performance characteristics (Figure S1) was not further improved with helper RNAs (Figure S2). One possible explanation is that the helper RNAs may not enhance the toehold sensor’s characteristics themselves but rather help present the target site with low accessibility. The mCherry fluorescence and *mCherry* mRNA amount was not particularly affected by the length or the position of helper RNAs (Figures S3 and S4), indicating that target mRNA translation was not affected and that this strategy could be utilized for sensing other important natural target RNAs. 

### 3.2. Automated Toehold Switch Design for clb ORFs in the pks Island

The *pks* island is a well-known pathogenicity island, which produces a genotoxin, colibactin. Recent studies revealed its various biological roles, such as probiotic activity [64,65], antibiotic activity [66], and analgesic lipopeptide production [67]. Therefore, repurposing toehold switch sensors to target genes within the *pks* island may provide a much-needed synthetic biological toolkit to characterize and control the diverse set of genetic elements within the *pks* island. 

We selected four essential genes for the *pks* island mRNA sensor development: *clbA*, *clbE*, *clbP*, and *clbQ*, among the 19 genes [53]. ClbA is involved at the beginning of the colibactin biosynthesis pathway [63], and its transcription level was increased in the stools of colorectal cancer patients [81]. ClbP contributes to the final maturation of pre-colibactin [82] and the antimicrobial activity of *E. coli* Nissle [65]. ClbQ mediates off-loading of several colibactin intermediates, and its inactivation leads to dramatic reductions [83]. Even though ClbE has not been explored in-depth, it was selected as a design target since its mRNA length was very short compared to other ORFs. The *clbE* was the shortest with an ORF of 249 bp while other selected ORFs, *clbA*, *clbP*, and *clbQ*, were 735 bp, 1515 bp, and 723 bp [76], respectively. A longer mRNA molecule could have more potential ways to form intramolecular secondary structures, which in turn can decrease accessibility and thermodynamical compensation for toehold switch sensors. The colibactin genes were obtained from *E. coli* Nissle 1917 and cloned under T7 promoter (pT7). 

**Figure 3:**
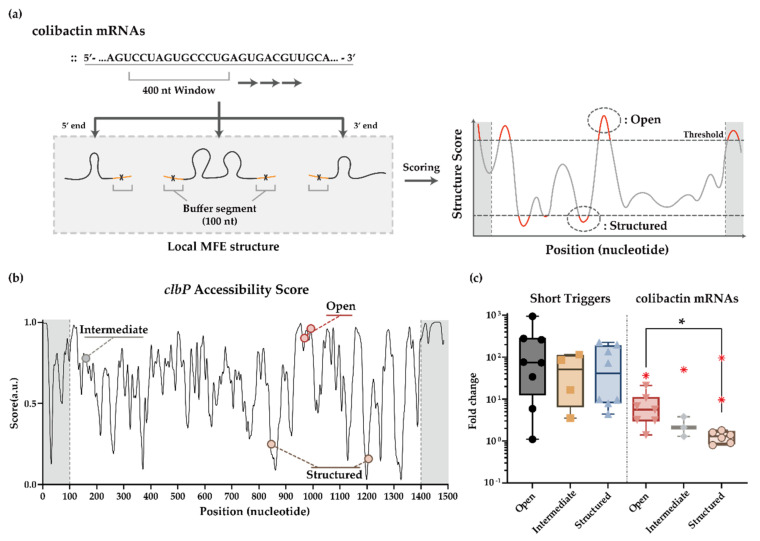
Automated target site selection algorithm and its application to *clb* sensor designs. (**a**) Brief scheme for NUPACK based target site selection algorithm. Structure score was evaluated based on the number of structure-free nucleotides within a 30 nt, size of a cognate trigger. (**b**) Accessibility evaluation result of full-length *clbP* mRNA. A high score indicates a high probability of single-strandedness. Curve smoothing was performed. (**c**) In vivo characterization of *clb* mRNA sensors. Synthetic short triggers and colibactin mRNAs were used as the cognate triggers. Fold change was calculated with ON/OFF GFP fluorescence ratios. GFP fluorescence was measured on flow cytometry. Exceptions of less than 1% in statistics were marked as red X. The exceptions were excluded for statistical analysis. (two-tailed Student’s *t*-test; * *p* < 0.05).

To help design toehold switch sensors for *clb* ORFs, we employed an automated screening process using the RNA structure prediction algorithm NUPACK [71,72,73,74,75]. First, we aimed to identify the regions within full-length mRNAs that are predicted to have little to no secondary structures. Since there is a relatively large uncertainty for the structure of full-length mRNAs, which can encompass up to >1 kb for our choices, we decided to focus on a smaller window of mRNA segments and assess the secondary structures in that window. We reasoned that this evaluation process might correlate with the cotranscriptional folding process of mRNA molecules as they are transcribed. Specifically, based on the local MFE structure in a window of 400 nt segment, accessibility was evaluated using the number of free nucleotides in the central 200 nt window (Figure 3a), repeating the process for every 30 nt step, the size of a cognate trigger for a toehold switch sensor (Figure 3a,b and Figure S5). In the case of *clbE* whose mRNA size is smaller than 400 nt, the local MFE structure was analyzed without choosing a smaller window. Second, toehold switch sensors were designed to target highly structured regions within *clb* mRNAs using the same automated screening process. Third, additional toehold switch sensors were designed that target sequence domain that is predicted to be neither highly accessible nor highly structured. Suppose the secondary structure of target domains plays an important role, and the evaluated MFE structure of RNAs correlates well with actual RNA folding dynamics. In that case, these automated screening and design processes could result in toehold switch sensors whose output characteristics correlate well with the predicted structure of target sites.

### 3.3. In Vivo Sensing of clb Genes Using Toehold Switch Sensors

To evaluate the functionality of *clb* mRNA sensors in the absence of potential secondary structures in the trigger RNA molecules, toehold switch sensors were transformed with a synthetic short trigger or a noncognate decoy RNA. The experiments were performed in *E. coli* BL21 AI strain with 0.2% arabinose (*w*/*v*) as inducers to produce genomically encoded T7 RNA polymerase. Afterward, GFP fluorescence was measured through flow cytometry, and fold changes of GFP outputs compared to a noncognate decoy RNA input were plotted (Figure 3c and Figure S6a). While *clb* mRNA sensors’ performance was highly variable, with some sensors showing close to three-orders-of-magnitude change upon triggering, there was no apparent trend for the three groups of *clb* mRNA sensors designed to target open, structured, and another randomly chosen region (named intermediate sensor). 

Still, sensing the full-length mRNAs may present challenges to the toehold switch sensors in vivo. To evaluate the sensing performance of *clb* mRNA sensors to its target, full-length mRNAs, cognate *clb* ORFs and *clb* mRNA sensors were co-transformed into *E. coli* BL21 AI strain. The fold changes of output GFP signals upon triggering were substantially reduced when compared with those obtained using synthetic short RNA inputs (Figure 3c). For the *clb* mRNA sensors targeting supposedly open target sites, the fold changes were median 6.2 fold with up to 36.4 fold at maximum. For the *clb* mRNA sensors that targeted regions predicted to be highly structured, the fold changes were close to 1.5 except for the two cases. Overall, the fold change of GFP outputs tended to be higher for the *clb* mRNA sensors that sense the regions predicted to be free of significant secondary structures (Figure S6b,c). Of note, we observed *clb* mRNA sensors that deviate from the overall trends, especially for the sensors that target *clbQ* mRNA (Figure S6c). Thus, we observed the overall trend as expected, but the exact outcome seemed to depend sensitively on the identity of the targeted genes and sequence domains. 

### 3.4. HAM System for the clb ORFs Targeting Toehold Switch Sensors

Despite the limited success of *clb* toehold sensors in response to its full-length mRNA inputs, the good performance of the same *clb* toehold sensors in detecting short synthetic RNAs indicates that the HAM system’s application could potentially boost the functionality of the *clb* mRNA sensors. To test the effect of the HAM systems, we applied helper RNAs around the target sequences that are supposed to be mostly free of secondary structures (open) or other random regions (intermediate). Highly structured region-targeting mRNA sensors were excluded due to a strong decline in full-length mRNA sensing capability for most sensor designs tested (Figure 3c). Among those that target structure-free domains (open), *clbA* and *clbE* sensors were not further investigated due to poor performance (*clbA* sensors, Figure S7) or their terminal locations (*clbE* sensors, Figure S6). Helper RNAs were designed to target 30 nt stretches upstream and downstream of a target site, and experimental characterizations were carried out in the same manner. 

**Figure 4:**
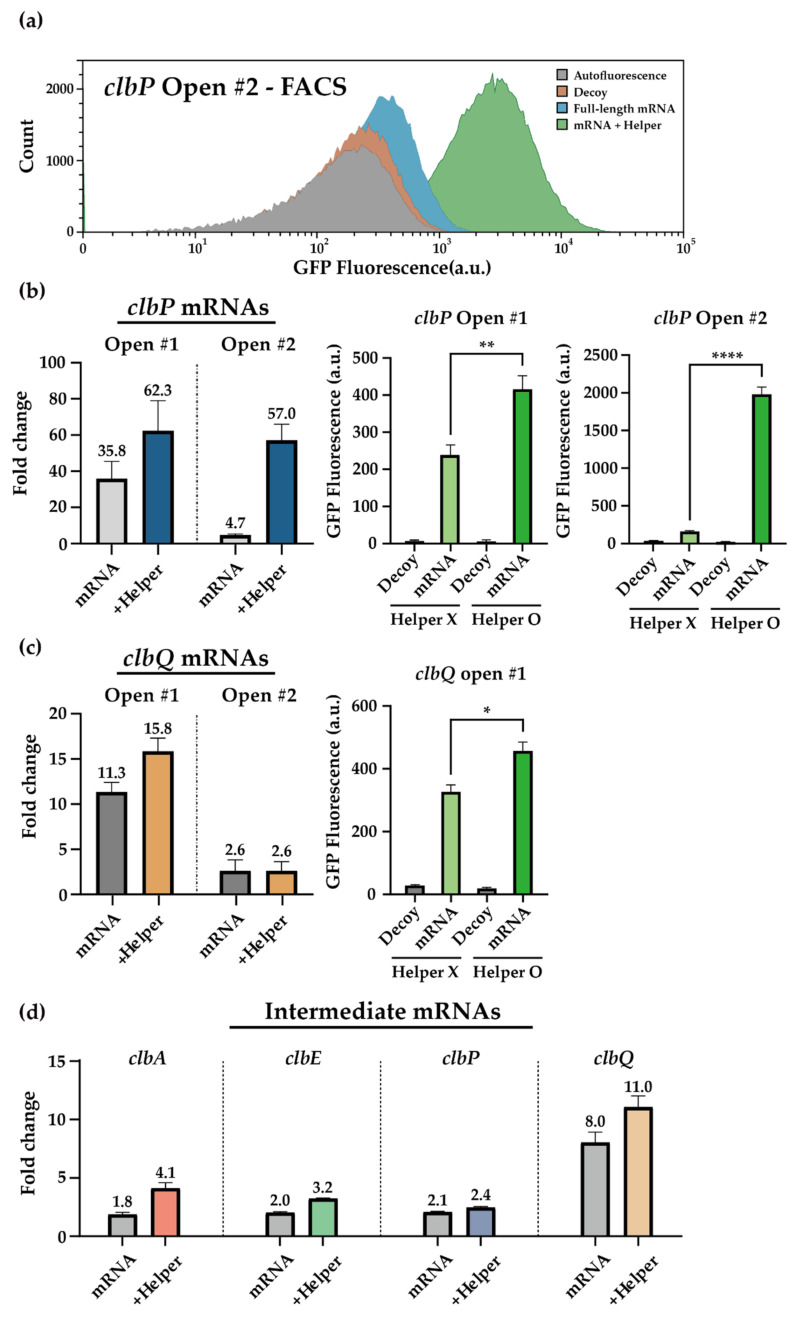
Helper-assisted mRNA sensing on Colibactin mRNAs. (**a**) Flow cytometry GFP fluorescence histograms for *clbP* open region targeting sensor 2 with or without helper RNA in the presence of cognate full-length trigger RNA or decoy RNA. Autofluorescence level was measured from cells not bearing a GFP-expressing plasmid. (**b**,**c**) Fold change enhancement of *clbP* and *clbQ* mRNA open region targeting sensors with helper RNAs. (two-tailed Student’s *t*-test; * *p* < 0.05; ** *p* < 0.01; **** *p* < 0.0001) (**d**) Fold change enhancement of *clbA*, *clbE*, *clbP*, and *clbQ* mRNA intermediate sensors in the presence of helper RNAs. Full-length mRNAs were used as the cognate trigger of sensors in all figures. Fold changes were calculated by dividing GFP fluorescence on cognate mRNA by those of decoy RNA. GFP fluorescence measurements were performed on flow cytometry (error bars indicate ± SEM).

Encouragingly, *clbP* and *clbQ* sensor outputs were improved in the presence of helper RNAs (Figure 4a–c and Figure S8). The greatest improvement was observed for the *clbP* open sensor 2, whose fold-changes improved from 4.7 folds without helper RNAs to 57.0 folds in the presence of helper RNAs. This improvement was achieved through statistically significant increases of the *clb* sensor ON levels (Figure 4b,c), while there was little change in the number of target mRNAs (Figure S9). The *clbQ* open sensor 2, however, was insensitive to the additional helper RNAs. The helper RNAs could improve *clbA* sensors to some extent (Figure S7) and similarly for the intermediate sensors (Figure 4d). The addition of the HAM system did not affect the sensing ability against short synthetic triggers (Figure S10). This indicated that the improved output signals in the presence of full-length mRNA inputs together with helper RNAs were likely due to the increased accessibility of target regions via interaction of helper and mRNAs. 

## 4. Discussion

Inspired by the natural versatility of RNA molecules, synthetic biologists have engineered RNA devices with novel biological functions. Especially promising is the suite of de-novo-designed synthetic RNA regulators that encompass multi-level regulation of genetic circuits involving transcription and translation processes and are broadly applicable to engineer scalable and programmable cellular behaviors as well as novel molecular diagnostics [21,84,85]. To further enhance the functionality of the de-novo-designed toehold switch library, we designed and implemented a HAM system to linearize target regions within a long mRNA species at the molecular level. The feasibility test of the HAM system using a previously characterized *mCherry* mRNA sensor could improve the output signals, suggesting that the helper RNAs could be employed to amplify signals in vivo. Of note, mCherry fluorescence and mRNA transcript level was not noticeably perturbed with the introduction of the helper RNAs, indicating that this strategy could be utilized for sensing other important natural target RNAs. 

Due to its intricate involvement in human pathology in the gut, the *pks* genomic island found in *E. coli* strains can serve as an important target for molecular diagnostics. To confirm the applicability of toehold switch sensors in conjunction with the HAM system, we designed toehold switch sensors targeting four essential ORFs in the *pks* island with their respective helper RNAs. We implemented an algorithm to screen for sites with a low probability of secondary structures within the target mRNAs to further enhance the probability of successful sensor construction. Overall, the functionality of toehold switch sensors correlated with the expected structure-free regions and could be further enhanced via helper RNAs. Using predicted MFE structures of mRNAs in the presence and absence of helper RNAs, we observed a reduced number of base pairs within the target domains in the presence of helper RNAs (Figure S11 and S12). In addition, mRNA and toehold sensor complex formations are more thermodynamically favorable in the presence of helper RNAs (Table S11). The *clb* mRNA sensors showed comparable performance to previously reported mRNA sensors, and helper RNAs could improve the performance of mRNA sensors (Table S12). These findings indicate that the combined application of the HAM system at the molecular level and the automated algorithm at the design level could help streamline the discovery process of highly functional toehold switches for these classes of long RNA targets. 

Despite the success, we also observed that the predictive power of current models is limited. For instance, *clbA* open sensor 1 and *clbE* open sensor 1 showed poor performance with 1.4-fold and 3.1-fold changes for full-length mRNA detection, respectively. A closer inspection revealed that these sensors showed high leakage levels in the absence of cognate triggers, possibly due to the low GC content at the base of the toehold switch lower stem (Figure S13). Since previous works indicated that the appropriate GC content in toehold switch variants is conducive to achieving wide dynamic ranges [19,22], the base composition can be factored in for further design iterations. On the other hand, *clbQ* structured sensor 2 and *clbA* structured sensor 2 showed decent performance of 75.8-fold and 9.0-fold changes, respectively. This may indicate that the RNA secondary structure prediction tools have certain limitations [86,87], in the sense that several alternative secondary structures can be predicted with similar thermodynamic energy levels (Figure S14). Another potential explanation for the limited predictive power of current thermodynamic models for RNA folding tasks concerns the influence of RNA folding kinetics that occur co-transcriptionally [88,89,90]. In yet another instance, the introduction of helper RNAs failed to further improve fold changes of sensor outputs, as in the case of *clbQ* open sensor 2, possibly due to the highly structured nature of helper binding domains (Figure S5). Since helper RNAs function by increasing the accessibility of target sites, it may not be so effective if the target site is already highly accessible or highly structured [49]. Together, additional optimization for helper RNA designs and target selection algorithms would be required to factor in the kinetic folding process, cellular burden, and molecular ratio of RNA components. Analogous to machine learning approaches for the large library of toehold switch designs [91,92], it would be possible to improve the design algorithms with sufficient high-quality data for toehold switch mRNA sensors. 

Our method for detecting relatively long RNA sequences with synthetic RNA sensors could be applied to a broad range of studies that require searching and stabilizing single-stranded regions of RNA. While previous works have employed RNA destabilizing chaperons [93] and helper oligonucleotide in vitro [47,48], we further improved the RNA sensing in vivo with the combined use of helper RNAs and automated design algorithms. These tools can not only help improve toehold switches and other similar RNA sensors but could also be useful for targeting and engineering cas13a mediated RNA knockdown [94], transcriptome engineering [95], alternative splicing [96], or trans-cleaving ribozyme mediated RNA processing and degradation [97,98]. Taken together, the improved sensing capability of toehold switches for an important class of natural RNA transcripts, when combined with its programmability, low crosstalk, and complex computational logic capability, could contribute to future developments of smart probiotics [99,100,101], in situ microbiome editing [34,35,36], and in vivo cellular RNA detection [19,22]. 

## Data Availability

The data presented in this study are available on request from the corresponding author.

## Supplementary Materials

The following are available online at https://www.mdpi.com/article/10.3390/life11111280/s1, Table S1: Plasmids used in this study. Table S2: Examples of DNA plasmid sequences. Table S3: *mCherry* mRNA sensor sequences used in this study. Table S4: Helper sequences for *mCherry* mRNA sensor used in this study. Table S5: *clb* mRNA sequences used in this study. Table S6: *clb* synthetic short trigger sequences used in this study. Table S7: *clb* mRNA sensor sequences used in this study. Table S8: Helper sequences for *clb* mRNA sensor used in this study. Table S9: Other accessory sequences used in this study. Table S10: Primers used for qPCR. Table S11: Gibbs free energy of mRNAs, mRNA sensors, and RNA complexes studied in this paper. Table S12: Performance information for a selection of toehold switches, toehold repressors, 3WJ repressors, and *clb* sensors. Figure S1: *mCherry* mRNA sensor variants tested in this study. Figure S2: Performance of *mCherry* sensor variant 1 with helper RNAs. Figure S3: mCherry expression levels in the presence of helper RNA variants. Figure S4: RT-qPCR data for *mCherry* mRNA levels. Figure S5: Accessibility calculation of *clbA*, *clbE*, *clbP*, and *clbQ*. Figure S6: In vivo performance of *clb* mRNA sensors. Figure S7: In vivo performance of *clbA* open sensors with helper RNAs. Figure S8: Flow cytometry data for *clbP* and *clbQ* open sensors with helper RNAs. Figure S9: RT-qPCR data for colibactin mRNA levels. Figure S10: In vivo performance of *clbP* and *clbQ* open sensors with short triggers and helper RNAs. Figure S11: Predicted MFE structures for *mCherry* mRNA without and with helper RNAs. Figure S12: Predicted MFE structures for *clbP* mRNAs without and with helper RNAs. Figure S13: The OFF-state fluorescence of *clb* sensors and the GC content of the lower stem. Figure S14: Predicted alternative secondary structures for *clbQ* and *clbA* mRNAs.
