# Supplementary material for: Detection of pks Island mRNAs Using Toehold Sensors in Escherichia coli
Source: Life (Basel). 2021 Nov 22;11(11):1280. doi: 10.3390/life11111280 (PMC8625898; doi:10.3390/life11111280)
Supplement: Supplementary file 1 [file life-11-01280-s001.zip › Life_SI_1123_corrected.pdf]

life

Supplementary

Detection of *pks* Island mRNAs Using Toehold Sensors in *Escherichia coli*

Taeyang Heo [1,†], Hansol Kang [1,†], Seungdo Choi [1,†], Jongmin Kim [1,*]

[1] Department of Life Sciences, Pohang University of Science and Technology, Pohang 37673, Korea; hty1998@postech.ac.kr (T.H.); hskang0405@postech.ac.kr (H.K.); choisd@postech.ac.kr (S.C.); jongmin.kim@postech.ac.kr (J.K.)
† Equal contribution: Taeyang Heo, Hansol Kang, Seungdo Choi
* Correspondence: jongmin.kim@postech.ac.kr

Detailed Experimental Protocols

Plasmid construction

Plasmids were constructed using circular polymerase extension cloning (CPEC) [1], Gibson assembly [2] and round-the-horn site-directed mutagenesis [3]. All DNA templates for helper-assisted mRNA sensing circuit and our algorithm-based mRNA sensor design were obtained by oligonucleotide synthesis. The synthetic DNA strands were PCR amplified and inserted into plasmid backbones. All plasmids were cloned in the E. coli DH5a strain and validated through DNA Sequencing. Backbones for the plasmids were taken from the commercial vectors pET15b, pCDFDuet, and pCOLADuet (EMD Millipore). GFPmut3b_ASV was used as the reporter [4], and the ASV degradation tag addition were done by round-the-horn site-directed mutagenesis. Sequences of elements commonly used in the plasmids are provided in Table S5-S9.

Microplate reader analysis

For the in vivo validation of *mCherry* mRNA sensors (Figure S1), 200 µL of cell cultures after 0.2 % arabinose (*w*/*v*) induction were added per well on a 96-well Black Plate 33396 (SPL). GFP fluorescence (excitation: 479 nm, emission: 520 nm), mCherry fluorescence (excitation: 587 nm, emission: 610 nm) and OD600 were measured at a Synergy H1 microplate reader (Biotek) running Gen5 3.08 software. GFP and mCherry fluorescence levels were normalized as follows: Fluorescence of LB blank was subtracted for background normalization, and a measured fluorescence value was divided by its OD600. The number of biological replicates was three for in vivo experiments.

Supplementary Tables

Supplementary Table S1. Plasmids used in this study. Abbreviations are as follows: pT7 = T7 promoter, T7term = T7 terminator, AmpR = ampicillin resistance gene, SpecR = spectinomycin resistance gene, kanR = kanamycin resistance gene. All the target mRNAs, sensor RNAs and helper RNAs were cloned into pET15b, pCOLADUET, pCDFDUET plasmid, respectively.

Name	Sequence
Cognate trigger	pT7–Cognate trigger–T7term-AmpR-pBR322 origin-LacI
Sensor	pT7-Sensor-Linker-GFPmut3b_ASV-T7term-kanR-ColA origin-LacI
Helper	pT7-Helper-T7term-SpecR-CloDF origin-LacI

Supplementary Table S2. Examples of DNA plasmid sequences. Replication origin and LacI was reversely oriented to other elements.

Name (architecture)	Sequence
mCherry mRNA (pT7–RBS-mCherry–T7term–(Bla Promoter)–AmpR–pBR322 origin–backbone-LacI-(Lac promoter))	TAATACGACTCACTATAGGGGAATTGTGAGCGGATAACAATTCCCCTCTAG<br>AAATAATTTTGTTTAACTTTAAGAAGGAGATATACCATGGGCAGCAGCCAT<br>CATCATCATCATCACAGCAGCGGCCTGGTGCCGCGCGGCAGCCATATGCGT<br>AAAATGGTGAGCAAGGGCGAAGAAGATAACATGGCCATCATCAAGGAGT<br>TCATGCGCTTCAAGGTTCACATGGAGGGCTCCGTGAACGGCCACGAGTTCG<br>AGATCGAGGGCGAGGGCGAGGGCCGCCCCTACGAGGGCACCCAGACCGC<br>CAAGCTGAAGGTGACCAAGGGTGGCCCCCTGCCCTTCGCCTGGGACATCCT<br>GTCCCCTCAGTTCATGTACGGCTCCAAGGCCTACGTGAAGCACCCCGCCGA<br>CATCCCCGACTACTTGAAGCTGTCCTTCCCCGAGGGCTTCAAGTGGGAGCG<br>CGTGATGAACTTCGAGGACGGCGGCGTGGTGACCGTGACCCAGGACTCCT<br>CCCTGCAAGACGGCGAGTTCATCTACAAGGTGAAGCTGCGCGGCACCAAC<br>TTCCCCTCCGACGGCCCCGTAATGCAGAAGAAGACTATGGGCTGGGAGGC<br>CTCCTCCGAGCGGATGTACCCCGAGGACGGCGCGCTGAAGGGCGAGATCA<br>AGCAGAGGCTGAAGCTGAAGGACGGCGGCCACTACGACGCTGAGGTCAA<br>GACCACCTACAAGGCCAAGAAGCCCGTGCAACTGCCCGGCGCGTACAACG<br>TCAACATCAAGTTGGACATCACCTCCCACAACGAGGACTACACCATCGTG<br>GAACAGTACGAACGCGCCGAGGGCCGCCACTCCACCGGCGGCATGGACG<br>AGCTGTACAAGTAACTAGCATAACCCCTTGGGGCCTCTAAACGGGTCTTGA<br>GGGGTTTTTTGCTGAAAGGAGGAACTATATCCGGATATCCCGCAAGAGGCC<br>CGGCAGTACCGGCATAACCAAGCCTATGCCTACAGCATCCAGGGTGACGG<br>TGCCGAGGATGACGATGAGCGCATTGTTAGATTTCATACACGGTGCCTGAC<br>TGCGTTAGCAATTTAACTGTGATAAACTACCGCATTAAAGCTTATCGATGA<br>TAAGCTGTCAAACATGAGAATTCTTGAAGACGAAAGGGCCTCGTGATACG<br>CCTATTTTTATAGGTTAATGTCATGATAATAATGGTTTCTTAGACGTCAGGT<br>GGCACTTTTCGGGGAAATGTGCGCGGAACCCCTATTTGTTTATTTTTCTAAA<br>TACATTCAAATATGTATCCGCTCATGAGACAATAACCCTGATAAATGCTTC<br>AATAATATTGAAAAAGGAAGAGTATGAGTATTCAACATTTCCGTGTCGCCC<br>TTATTCCCTTTTTTGCGGCATTTTGCCTTCCTGTTTTTGCTCACCCAGAAACG<br>CTGGTGAAAGTAAAAGATGCTGAAGATCAGTTGGGTGCACGAGTGGGTTA<br>CATCGAACTGGATCTCAACAGCGGTAAGATCCTTGAGAGTTTTCGCCCCGA<br>AGAACGTTTTCCAATGATGAGCACTTTTAAAGTTCTGCTATGTGGCGCGGT<br>ATTATCCCGTGTTGACGCCGGGCAAGAGCAACTCGGTCGCCGCATACACTA<br>TTCTCAGAATGACTTGGTTGAGTACTCACCAGTCACAGAAAAGCATCTTAC<br>GGATGGCATGACAGTAAGAGAATTATGCAGTGCTGCCATAACCATGAGTG<br>ATAACACTGCGGCCAACTTACTTCTGACAACGATCGGAGGACCGAAGGAG<br>CTAACCGCTTTTTTGCACAACATGGGGGATCATGTAACTCGCCTTGATCGTT<br>GGGAACCGGAGCTGAATGAAGCCATACCAAACGACGAGCGTGACACCAC

	GATGCCTGCAGCAATGGCAACAACGTTGCGCAAACTATTAACTGGCGAAC<br>TACTTACTCTAGCTTCCCGGCAACAATTAATAGACTGGATGGAGGCGGATA<br>AAGTTGCAGGACCACTTCTGCGCTCGGCCCTTCCGGCTGGCTGGTTTATTGC<br>TGATAAATCTGGAGCCGGTGAGCGTGGGTCTCGCGGTATCATTGCAGCACT<br>GGGGCCAGATGGTAAGCCCTCCCGTATCGTAGTTATCTACACGACGGGGA<br>GTCAGGCAACTATGGATGAACGAAATAGACAGATCGCTGAGATAGGTGCC<br>TCACTGATTAAGCATTGGTAACTGTCAGACCAAGTTTACTCATATATACTTT<br>AGATTGATTTAAAACTTCATTTTTAATTTAAAAGGATCTAGGTGAAGATCCT<br>TTTTGATAATCTCATGACCAAAATCCCTTAACGTGAGTTTTCGTTCCACTGA<br>GCGTCAGACCCCGTAGAAAAGATCAAAGGATCTTCTTGAGATCCTTTTTTT<br>CTGCGCGTAATCTGCTGCTTGCAAACAAAAAAACCACCGCTACCAGCGGT<br>GGTTTGTTTGCCGGATCAAGAGCTACCAACTCTTTTTCCGAAGGTAACTGGC<br>TTCAGCAGAGCGCAGATACCAAATACTGTCCTTCTAGTGTAGCCGTAGTTA<br>GGCCACCACTTCAAGAACTCTGTAGCACCGCCTACATACCTCGCTCTGCTA<br>ATCCTGTTACCAGTGGCTGCTGCCAGTGGCGATAAGTCGTGTCTTACCGGG<br>TTGGACTCAAGACGATAGTTACCGGATAAGGCGCAGCGGTCGGGCTGAAC<br>GGGGGGTTCGTGCACACAGCCCAGCTTGGAGCGAACGACCTACACCGAAC<br>TGAGATACCTACAGCGTGAGCTATGAGAAAGCGCCACGCTTCCCGAAGGG<br>AGAAAGGCGGACAGGTATCCGGTAAGCGGCAGGGTCGGAACAGGAGAGC<br>GCACGAGGGAGCTTCCAGGGGGAAACGCCTGGTATCTTTATAGTCCTGTCG<br>GGTTTCGCCACCTCTGACTTGAGCGTCGATTTTTGTGATGCTCGTCAGGGGG<br>GCGGAGCCTATGGAAAAACGCCAGCAACGCGGCCTTTTTACGGTTCCTGGC<br>CTTTTGCTGGCCTTTTGCTCACATGTTCTTTCCTGCGTTATCCCCTGATTCTGT<br>GGATAACCGTATTACCGCCTTTGAGTGAGCTGATACCGCTCGCCGCAGCCG<br>AACGACCGAGCGCAGCGAGTCAGTGAGCGAGGAAGCGGAAGAGCGCCTG<br>ATGCGGTATTTTCTCCTTACGCATCTGTGCGGTATTTCACACCGCATATATG<br>GTGCACTCTCAGTACAATCTGCTCTGATGCCGCATAGTTAAGCCAGTATAC<br>ACTCCGCTATCGCTACGTGACTGGGTCATGGCTGCGCCCCGACACCCGCCA<br>ACACCCGCTGACGCGCCCTGACGGGCTTGTCTGCTCCCGGCATCCGCTTAC<br>AGACAAGCTGTGACCGTCTCCGGGAGCTGCATGTGTCAGAGGTTTTCACCG<br>TCATCACCGAAACGCGCGAGGCAGCTGCGGTAAAGCTCATCAGCGTGGTC<br>GTGAAGCGATTCACAGATGTCTGCCTGTTCATCCGCGTCCAGCTCGTTGAGT<br>TTCTCCAGAAGCGTTAATGTCTGGCTTCTGATAAAGCGGGCCATGTTAAGG<br>GCGGTTTTTTCCTGTTTGGTCACTGATGCCTCCGTGTAAGGGGGATTTCTGTT<br>CATGGGGGTAATGATACCGATGAAACGAGAGAGGATGCTCACGATACGGG<br>TTACTGATGATGAACATGCCCGGTTACTGGAACGTTGTGAGGGTAAACAAC<br>TGGCGGTATGGATGCGGCGGGACCAGAGAAAAATCACTCAGGGTCAATGC<br>CAGCGCTTCGTTAATACAGATGTAGGTGTTCCACAGGGTAGCCAGCAGCAT<br>CCTGCGATGCAGATCCGGAACATAATGGTGCAGGGCGCTGACTTCCGCGTT<br>TCCAGACTTTACGAAACACGGAAACCGAAGACCATTCATGTTGTTGCTCAG<br>GTCGCAGACGTTTTGCAGCAGCAGTCGCTTCACGTTCGCTCGCGTATCGGT<br>GATTCATTCTGCTAACCAGTAAGGCAACCCCGCCAGCCTAGCCGGGTCCTC<br>AACGACAGGAGCACGATCATGCGCACCCGTGGCCAGGACCCAACGCTGCC<br>CGAGATGCGCCGCGTGCGGCTGCTGGAGATGGCGGACGCGATGGATATGT<br>TCTGCCAAGGGTTGGTTTGCGCATTCACAGTTCTCCGCAAGAATTGATTGGC<br>TCCAATTCTTGGAGTGGTGAATCCGTTAGCGAGGTGCCGCCGGCTTCCATTC<br>AGGTCGAGGTGGCCCGGCTCCATGCACCGCGACGCAACGCGGGGAGGCAG<br>ACAAGGTATAGGGCGGCGCCTACAATCCATGCCAACCCGTTCCATGTGCTC<br>GCCGAGGCGGCATAAATCGCCGTGACGATCAGCGGTCCAGTGATCGAAGT<br>TAGGCTGGTAAGAGCCGCGAGCGATCCTTGAAGCTGTCCCTGATGGTCGTC<br>ATCTACCTGCCTGGACAGCATGGCCTGCAACGCGGGCATCCCGATGCCGCC

	GGAAGCGAGAAGAATCATAATGGGGAAGGCCATCCAGCCTCGCGTCGCGA<br>ACGCCAGCAAGACGTAGCCCAGCGCGTCGGCCGCCATGCCGGCGATAATG<br>GCCTGCTTCTCGCCGAAACGTTTGGTGGCGGGACCAGTGACGAAGGCTTGA<br>GCGAGGGCGTGCAAGATTCCGAATACCGCAAGCGACAGGCCGATCATCGT<br>CGCGCTCCAGCGAAAGCGGTCCTCGCCGAAAATGACCCAGAGCGCTGCCG<br>GCACCTGTCCTACGAGTTGCATGATAAAGAAGACAGTCATAAGTGCGGCG<br>ACGATAGTCATGCCCCGCGCCCACCGGAAGGAGCTGACTGGGTTGAAGGC<br>TCTCAAGGGCATCGGTCGAGATCCCGGTGCCTAATGAGTGAGCTAACTTAC<br>ATTAATTGCGTTGCGCTCACTGCCCGCTTTCCAGTCGGGAAACCTGTCGTGC<br>CAGCTGCATTAATGAATCGGCCAACGCGCGGGGAGAGGCGGTTTGCGTAT<br>TGGGCGCCAGGGTGGTTTTTCTTTTCACCAGTGAGACGGGCAACAGCTGAT<br>TGCCCTTCACCGCCTGGCCCTGAGAGAGTTGCAGCAAGCGGTCCACGCTGG<br>TTTGCCCCAGCAGGCGAAAATCCTGTTTGATGGTGGTTAACGGCGGGATAT<br>AACATGAGCTGTCTTCGGTATCGTCGTATCCCACTACCGAGATATCCGCAC<br>CAACGCGCAGCCCGGACTCGGTAATGGCGCGCATTGCGCCCAGCGCCATC<br>TGATCGTTGGCAACCAGCATCGCAGTGGGAACGATGCCCTCATTCAGCATT<br>TGCATGGTTTGTTGAAAACCGGACATGGCACTCCAGTCGCCTTCCCGTTCC<br>GCTATCGGCTGAATTTGATTGCGAGTGAGATATTTATGCCAGCCAGCCAGA<br>CGCAGACGCGCCGAGACAGAACTTAATGGGCCCGCTAACAGCGCGATTTG<br>CTGGTGACCCAATGCGACCAGATGCTCCACGCCCAGTCGCGTACCGTCTTC<br>ATGGGAGAAAATAATACTGTTGATGGGTGTCTGGTCAGAGACATCAAGAA<br>ATAACGCCGGAACATTAGTGCAGGCAGCTTCCACAGCAATGGCATCCTGG<br>TCATCCAGCGGATAGTTAATGATCAGCCCACTGACGCGTTGCGCGAGAAG<br>ATTGTGCACCGCCGCTTTACAGGCTTCGACGCCGCTTCGTTCTACCATCGAC<br>ACCACCACGCTGGCACCCAGTTGATCGGCGCGAGATTTAATCGCCGCGAC<br>AATTTGCGACGGCGCGTGCAGGGCCAGACTGGAGGTGGCAACGCCAATCA<br>GCAACGACTGTTTGCCCGCCAGTTGTTGTGCCACGCGGTTGGGAATGTAAT<br>TCAGCTCCGCCATCGCCGCTTCCACTTTTTCCCGCGTTTTCGCAGAAACGTG<br>GCTGGCCTGGTTCACCACGCGGGAAACGGTCTGATAAGAGACACCGGCAT<br>ACTCTGCGACATCGTATAACGTTACTGGTTTCACATTCACCACCCTGAATTG<br>ACTCTCTTCCGGGCGCTATCATGCCATACCGCGAAAGGTTTTGCGCCATTCG<br>ATGGTGTCCGGGATCTCGACGCTCTCCCTTATGCGACTCCTGCATTAGGAA<br>GCAGCCCAGTAGTAGGTTGAGGCCGTTGAGCACCGCCGCCGCAAGGAATG<br>GTGCATGCAAGGAGATGGCGCCCAACAGTCCCCCGGCCACGGGGCCTGCC<br>ACCATACCCACGCCGAAACAAGCGCTCATGAGCCCGAAGTGGCGAGCCCG<br>ATCTTCCCCATCGGTGATGTCCGCGAAAT
Sensor RNA (pT7–Sensor C(RBS)–Linker–GFPmut3b_ASV–T7term–kanR–(Bla Promoter)–ColA origin-backbone-LacI-(Lac promoter))	TAATACGACTCACTATAGGGTGTGGGAGGTGATGTCCAACTTGATGTTGAC<br>GTTGTGTTATAGTTATGAGACAAGAACAGAGGAGACATAACATGAACACA<br>ACGAACTGTGTTAACCTGGCGGCAGCGCAAAAGATGCGTAAAGGAGAAG<br>AACTTTTCACTGGAGTTGTCCCAATTCTTGTTGAATTAGATGGTGATGTTAA<br>TGGGCACAAATTTTCTGTCAGTGGAGAGGGTGAAGGTGATGCAACATACG<br>GAAAACTTACCCTTAAATTTATTTGCACTACTGGAAAACTACCTGTTCCGTG<br>GCCAACACTTGTCACTACTTTCGGTTATGGTGTTCAATGCTTTGCGAGATAC<br>CCAGATCACATGAAACAGCATGACTTTTTCAAGAGTGCCATGCCCGAAGGT<br>TACGTACAGGAAAGAACTATATTTTTCAAAGATGACGGGAACTACAAGAC<br>ACGTGCTGAAGTCAAGTTTGAAGGTGATACCCTTGTTAATAGAATCGAGTT<br>AAAAGGTATTGATTTTAAAGAAGATGGAAACATTCTTGGACACAAATTGG<br>AATACAACTATAACTCACACAATGTATACATCATGGCAGACAAACAAAAG<br>AATGGAATCAAAGTTAACTTCAAAATTAGACACAACATTGAAGATGGAAG<br>CGTTCAACTAGCAGACCATTATCAACAAAATACTCCGATTGGCGATGGCCC<br>TGTCCTTTTACCAGACAACCATTACCTGTCCACACAATCTGCCCTTTCGAAA

	GATCCCAACGAAAAGAGAGACCACATGGTCCTTCTTGAGTTTGTAACCGCT<br>GCTGGGATTACACATGGCATGGATGAACTATACAAAAGGCCTGCAGCAAA<br>CGACGAAAACTACGCTGCATCAGTTTAATAAGATAAACCAGAGCGGCACG<br>GCAAGCAGAGTATACGAGATTCGGTAGCCACCGCTGAGCAATAACTAGCA<br>TAACCCCTTGGGGCCTCTAAACGGGTCTTGAGGGGTTTTTTGCTGAAACCTC<br>AGGCATTTGAGAAGCACACGGTCACACTGCTTCCGGTAGTCAATAAACCG<br>GTAAACCAGCAATAGACATAAGCGGCTATTTAACGACCCTGCCCTGAACC<br>GACGACAAGCTGACGACCGGGTCTCCGCAAGTGGCACTTTTCGGGGAAAT<br>GTGCGCGGAACCCCTATTTGTTTATTTTTCTAAATACATTCAAATATGTATC<br>CGCTCATGAATTAATTCTTAGAAAAACTCATCGAGCATCAAATGAAACTGC<br>AATTTATTCATATCAGGATTATCAATACCATATTTTTGAAAAAGCCGTTTCT<br>GTAATGAAGGAGAAAACTCACCGAGGCAGTTCCATAGGATGGCAAGATCC<br>TGGTATCGGTCTGCGATTCCGACTCGTCCAACATCAATACAACCTATTAATT<br>TCCCCTCGTCAAAAATAAGGTTATCAAGTGAGAAATCACCATGAGTGACG<br>ACTGAATCCGGTGAGAATGGCAAAAGTTTATGCATTTCTTTCCAGACTTGTT<br>CAACAGGCCAGCCATTACGCTCGTCATCAAAATCACTCGCATCAACCAAA<br>CCGTTATTCATTCGTGATTGCGCCTGAGCGAGACGAAATACGCGGTCGCTG<br>TTAAAAGGACAATTACAAACAGGAATCGAATGCAACCGGCGCAGGAACA<br>CTGCCAGCGCATCAACAATATTTTCACCTGAATCAGGATATTCTTCTAATAC<br>CTGGAATGCTGTTTTCCCGGGGATCGCAGTGGTGAGTAACCATGCATCATC<br>AGGAGTACGGATAAAATGCTTGATGGTCGGAAGAGGCATAAATTCCGTCA<br>GCCAGTTTAGTCTGACCATCTCATCTGTAACATCATTGGCAACGCTACCTTT<br>GCCATGTTTCAGAAACAACTCTGGCGCATCGGGCTTCCCATACAATCGATA<br>GATTGTCGCACCTGATTGCCCGACATTATCGCGAGCCCATTTATACCCATAT<br>AAATCAGCATCCATGTTGGAATTTAATCGCGGCCTAGAGCAAGACGTTTCC<br>CGTTGAATATGGCTCATACTCTTCCTTTTTCAATATTATTGAAGCATTTATCA<br>GGGTTATTGTCTCATGAGCGGATACATATTTGAATGTATTTAGAAAAATAA<br>ACAAATAGGCATGCTAGCGCAGAAACGTCCTAGAAGATGCCAGGAGGAT<br>ACTTAGCAGAGAGACAATAAGGCCGGAGCGAAGCCGTTTTTCCATAGGCT<br>CCGCCCCCCTGACGAACATCACGAAATCTGACGCTCAAATCAGTGGTGGC<br>GAAACCCGACAGGACTATAAAGATACCAGGCGTTTCCCCCTGATGGCTCC<br>CTCTTGCGCTCTCCTGTTCCCGTCCTGCGGCGTCCGTGTTGTGGTGGAGGCT<br>TTACCCAAATCACCACGTCCCGTTCCGTGTAGACAGTTCGCTCCAAGCTGG<br>GCTGTGTGCAAGAACCCCCCGTTCAGCCCGACTGCTGCGCCTTATCCGGTA<br>ACTATCATCTTGAGTCCAACCCGGAAAGACACGACAAAACGCCACTGGCA<br>GCAGCCATTGGTAACTGAGAATTAGTGGATTTAGATATCGAGAGTCTTGAA<br>GTGGTGGCCTAACAGAGGCTACACTGAAAGGACAGTATTTGGTATCTGCGC<br>TCCACTAAAGCCAGTTACCAGGTTAAGCAGTTCCCCAACTGACTTAACCTT<br>CGATCAAACCGCCTCCCCAGGCGGTTTTTTCGTTTACAGAGCAGGAGATTA<br>CGACGATCGTAAAAGGATCTCAAGAAGATCCTTTACGGATTCCCGACACC<br>ATCACTCTAGATTTCAGTGCAATTTATCTCTTCAAATGTAGCACCTGAAGTC<br>AGCCCCATACGATATAAGTTGTAATTCTCATGTTAGTCATGCCCCGCGCCC<br>ACCGGAAGGAGCTGACTGGGTTGAAGGCTCTCAAGGGCATCGGTCGAGAT<br>CCCGGTGCCTAATGAGTGAGCTAACTTACATTAATTGCGTTGCGCTCACTG<br>CCCGCTTTCCAGTCGGGAAACCTGTCGTGCCAGCTGCATTAATGAATCGGC<br>CAACGCGCGGGGAGAGGCGGTTTGCGTATTGGGCGCCAGGGTGGTTTTTCT<br>TTTCACCAGTGAGACGGGCAACAGCTGATTGCCCTTCACCGCCTGGCCCTG<br>AGAGAGTTGCAGCAAGCGGTCCACGCTGGTTTGCCCCAGCAGGCGAAAAT<br>CCTGTTTGATGGTGGTTAACGGCGGGATATAACATGAGCTGTCTTCGGTATC<br>GTCGTATCCCACTACCGAGATGTCCGCACCAACGCGCAGCCCGGACTCGGT<br>AATGGCGCGCATTGCGCCCAGCGCCATCTGATCGTTGGCAACCAGCATCGC

	AGTGGGAACGATGCCCTCATTCAGCATTTGCATGGTTTGTTGAAAACCGGA<br>CATGGCACTCCAGTCGCCTTCCCGTTCCGCTATCGGCTGAATTTGATTGCGA<br>GTGAGATATTTATGCCAGCCAGCCAGACGCAGACGCGCCGAGACAGAACT<br>TAATGGGCCCGCTAACAGCGCGATTTGCTGGTGACCCAATGCGACCAGAT<br>GCTCCACGCCCAGTCGCGTACCGTCTTCATGGGAGAAAATAATACTGTTGA<br>TGGGTGTCTGGTCAGAGACATCAAGAAATAACGCCGGAACATTAGTGCAG<br>GCAGCTTCCACAGCAATGGCATCCTGGTCATCCAGCGGATAGTTAATGATC<br>AGCCCACTGACGCGTTGCGCGAGAAGATTGTGCACCGCCGCTTTACAGGCT<br>TCGACGCCGCTTCGTTCTACCATCGACACCACCACGCTGGCACCCAGTTGA<br>TCGGCGCGAGATTTAATCGCCGCGACAATTTGCGACGGCGCGTGCAGGGC<br>CAGACTGGAGGTGGCAACGCCAATCAGCAACGACTGTTTGCCCGCCAGTT<br>GTTGTGCCACGCGGTTGGGAATGTAATTCAGCTCCGCCATCGCCGCTTCCA<br>CTTTTTCCCGCGTTTTCGCAGAAACGTGGCTGGCCTGGTTCACCACGCGGGA<br>AACGGTCTGATAAGAGACACCGGCATACTCTGCGACATCGTATAACGTTAC<br>TGGTTTCACATTCACCACCCTGAATTGACTCTCTTCCGGGCGCTATCATGCC<br>ATACCGCGAAAGGTTTTGCGCCATTCGATGGTGTCCGGGATCTCGACGCTC<br>TCCCTTATGAAGTCTAACGCTGCTCTGGGCTAACTGTC
Helper RNA (pT7–Helper2–T7term–pT7–Helper1–T7term–SpecR–(Bla Promoter)–CloDF13 origin–backbone- LacI-(Lac promoter))	TAATACGACTCACTATAGGGGGACCGGTCTTTGCAGAGACCGGTTGCCGCC<br>GGTGGAGTGGCGGCCCTCGGCGCGGGGTACTGTTCCACGATGGTGTAGTC<br>CTACTTAGCATAACCCCTTGGGGCCTCTAAACGGGTCTTGAGGGGTTTTTG<br>CTGAAACCTCAGGCATTTGAGAAGCACACGGTCACACTGCTTCCGGTAGTC<br>AATAAATAATACGACTCACTATAGGGCGTGAGATAAGCACATCTCACGTG<br>TACGCGCCGGGCAGTTGCACGGGCTTCTTAAGCTTGTAGGTGGTCTTGACCT<br>CAGCGACTTAGCATAACCCCTTGGGGCCTCTAAACGGGTCTTGAGGGGTTT<br>TTTGCCGGTAAACCAGCAATAGACATAAGCGGCTATTTAACGACCCTGCCC<br>TGAACCGACGACCGGGTCATCGTGGCCGGATCTTGCGGCCCCTCGGCTTGA<br>ACGAATTGTTAGACATTATTTGCCGACTACCTTGGTGATCTCGCCTTTCACG<br>TAGTGGACAAATTCTTCCAACTGATCTGCGCGCGAGGCCAAGCGATCTTCT<br>TCTTGTCCAAGATAAGCCTGTCTAGCTTCAAGTATGACGGGCTGATACTGG<br>GCCGGCAGGCGCTCCATTGCCCAGTCGGCAGCGACATCCTTCGGCGCGATT<br>TTGCCGGTTACTGCGCTGTACCAAATGCGGGACAACGTAAGCACTACATTT<br>CGCTCATCGCCAGCCCAGTCGGGCGGCGAGTTCCATAGCGTTAAGGTTTCA<br>TTTAGCGCCTCAAATAGATCCTGTTCAGGAACCGGATCAAAGAGTTCCTCC<br>GCCGCTGGACCTACCAAGGCAACGCTATGTTCTCTTGCTTTTGTCAGCAAG<br>ATAGCCAGATCAATGTCGATCGTGGCTGGCTCGAAGATACCTGCAAGAAT<br>GTCATTGCGCTGCCATTCTCCAAATTGCAGTTCGCGCTTAGCTGGATAACGC<br>CACGGAATGATGTCGTCGTGCACAACAATGGTGACTTCTACAGCGCGGAG<br>AATCTCGCTCTCTCCAGGGGAAGCCGAAGTTTCCAAAAGGTCGTTGATCAA<br>AGCTCGCCGCGTTGTTTCATCAAGCCTTACGGTCACCGTAACCAGCAAATC<br>AATATCACTGTGTGGCTTCAGGCCGCCATCCACTGCGGAGCCGTACAAATG<br>TACGGCCAGCAACGTCGGTTCGAGATGGCGCTCGATGACGCCAACTACCTC<br>TGATAGTTGAGTCGATACTTCGGCGATCACCGCTTCCCTCATACTCTTCCTT<br>TTTCAATATTATTGAAGCATTTATCAGGGTTATTGTCTCATGAGCGGATACA<br>TATTTGAATGTATTTAGAAAAATAAACAAATAGCTAGCTCACTCGGTCGCT<br>ACGCTCCGGGCGTGAGACTGCGGCGGGCGCTGCGGACACATACAAAGTTA<br>CCCACAGATTCCGTGGATAAGCAGGGGACTAACATGTGAGGCAAAACAGC<br>AGGGCCGCGCCGGTGGCGTTTTTCCATAGGCTCCGCCCTCCTGCCAGAGTT<br>CACATAAACAGACGCTTTTCCGGTGCATCTGTGGGAGCCGTGAGGCTCAAC<br>CATGAATCTGACAGTACGGGCGAAACCCGACAGGACTTAAAGATCCCCAC<br>CGTTTCCGGCGGGTCGCTCCCTCTTGCGCTCTCCTGTTCCGACCCTGCCGTTT<br>ACCGGATACCTGTTCCGCCTTTCTCCCTTACGGGAAGTGTGGCGCTTTCTCA

	TAGCTCACACACTGGTATCTCGGCTCGGTGTAGGTCGTTCGCTCCAAGCTG<br>GGCTGTAAGCAAGAACTCCCCGTTCAGCCCGACTGCTGCGCCTTATCCGGT<br>AACTGTTCACTTGAGTCCAACCCGGAAAAGCACGGTAAAACGCCACTGGC<br>AGCAGCCATTGGTAACTGGGAGTTCGCAGAGGATTTGTTTAGCTAAACACG<br>CGGTTGCTCTTGAAGTGTGCGCCAAAGTCCGGCTACACTGGAAGGACAGAT<br>TTGGTTGCTGTGCTCTGCGAAAGCCAGTTACCACGGTTAAGCAGTTCCCCA<br>ACTGACTTAACCTTCGATCAAACCACCTCCCCAGGTGGTTTTTTCGTTTACA<br>GGGCAAAAGATTACGCGCAGAAAAAAAGGATCTCAAGAAGATCCTTTGAT<br>CTTTTCTACTGAACCGCTCTAGATTTCAGTGCAATTTATCTCTTCAAATGTA<br>GCACCTGAAGTCAGCCCCATACGATATAAGTTGTAATTCTCATGTTAGTCA<br>TGCCCCGCGCCCACCGGAAGGAGCTGACTGGGTTGAAGGCTCTCAAGGGC<br>ATCGGTCGAGATCCCGGTGCCTAATGAGTGAGCTAACTTACATTAATTGCG<br>TTGCGCTCACTGCCCGCTTTCCAGTCGGGAAACCTGTCGTGCCAGCTGCATT<br>AATGAATCGGCCAACGCGCGGGGAGAGGCGGTTTGCGTATTGGGCGCCAG<br>GGTGGTTTTTCTTTTCACCAGTGAGACGGGCAACAGCTGATTGCCCTTCACC<br>GCCTGGCCCTGAGAGAGTTGCAGCAAGCGGTCCACGCTGGTTTGCCCCAGC<br>AGGCGAAAATCCTGTTTGATGGTGGTTAACGGCGGGATATAACATGAGCTG<br>TCTTCGGTATCGTCGTATCCCACTACCGAGATGTCCGCACCAACGCGCAGC<br>CCGGACTCGGTAATGGCGCGCATTGCGCCCAGCGCCATCTGATCGTTGGCA<br>ACCAGCATCGCAGTGGGAACGATGCCCTCATTCAGCATTTGCATGGTTTGT<br>TGAAAACCGGACATGGCACTCCAGTCGCCTTCCCGTTCCGCTATCGGCTGA<br>ATTTGATTGCGAGTGAGATATTTATGCCAGCCAGCCAGACGCAGACGCGCC<br>GAGACAGAACTTAATGGGCCCGCTAACAGCGCGATTTGCTGGTGACCCAA<br>TGCGACCAGATGCTCCACGCCCAGTCGCGTACCGTCTTCATGGGAGAAAAT<br>AATACTGTTGATGGGTGTCTGGTCAGAGACATCAAGAAATAACGCCGGAA<br>CATTAGTGCAGGCAGCTTCCACAGCAATGGCATCCTGGTCATCCAGCGGAT<br>AGTTAATGATCAGCCCACTGACGCGTTGCGCGAGAAGATTGTGCACCGCC<br>GCTTTACAGGCTTCGACGCCGCTTCGTTCTACCATCGACACCACCACGCTG<br>GCACCCAGTTGATCGGCGCGAGATTTAATCGCCGCGACAATTTGCGACGGC<br>GCGTGCAGGGCCAGACTGGAGGTGGCAACGCCAATCAGCAACGACTGTTT<br>GCCCGCCAGTTGTTGTGCCACGCGGTTGGGAATGTAATTCAGCTCCGCCAT<br>CGCCGCTTCCACTTTTTCCCGCGTTTTCGCAGAAACGTGGCTGGCCTGGTTC<br>ACCACGCGGGAAACGGTCTGATAAGAGACACCGGCATACTCTGCGACATC<br>GTATAACGTTACTGGTTTCACATTCACCACCCTGAATTGACTCTCTTCCGGG<br>CGCTATCATGCCATACCGCGAAAGGTTTTGCGCCATTCGATGGTGTCCGGG<br>ATCTCGACGCTCTCCCTTATGAGTGATAGCCGTTTGTCTGGTGTCTACGCCG<br>CGCGGGCTAACTGTC

Supplementary Table S3. *mCherry* mRNA sensor sequences used in this study. Common sequence element from high-performance first-generation toehold switch was colored in yellow [3]. Conserved linker sequence was indicated as gray. Trigger binding region was covered in green and its complementary region was colored in blue. Plasmid sequences of *mCherry* sensor can be constructed by replacing the sensor RNA region in the example plasmids in Table S2 with the *mCherry* sensor sequences described underneath.

Name	Target site	Sequence
Sensor A	448:477	AGGGGAAGTTGGTGCCGCGCAGCTTCACGTTATAGTTATGAGACAAGAACAGAGGAGACATAACATGAACGTGAAGAACCACGTTAACCTGGCGGCAGCGCAAAAG
Sensor C	662:697	TGTGGGAGGTGATGTCCAACTTGATGTTGACGTTGTGTTATAGTTATGAGACAAGAACAGAGGAGACATAACATGAACACAACGAACTGTGTTAACCTGGCGGCAGCGCAAAAG
Sensor Variant 1	568:602	TAGTGGCCGCCGTCCTTCAGCTTCAGCCTCTGCTTGTTATAGTTATGAGACAAGAACAGAGGAGACATAACATGAACAAGCAGAACCTTGTTAACCTGGCGGCAGCGCAAAAG
Sensor Variant 2	463:498	CTTCTGCATTACGGGGCCGTCGGAGGGGAAGTTGGTGTTATAGTTATGAGACAAGAACAGAGGAGACATAACATGAACACCAACAACGGTGTTAACCTGGCGGCAGCGCAAAAG

Supplementary Table S4. Helper sequences for *mCherry* mRNA sensor used in this study. Plasmid sequences of *mCherry* helper can be constructed by replacing the Helper 1 and Helper 2 region in the example plasmids in Table S2 with the appropriate helper sequences described underneath. 5′ structure was colored in green. mRNA binding regions were colored in yellow, and 3 nt bulge was colored in gray.

Name	Target site	Position	Sequence
Helper C			
H1-60	599:628 + 629:658	Upstream	CGTGAGATAAGCACATCTCACGCGCCGGGCAGTTGCACGGGCTTCTTGGCCTACATGTAGGTGGTCTTGACCTCAGCGTCGTAGT
H1-30	701:730 + 731:760	Upstream	CGTGAGATAAGCACATCTCACGCGCCGGGCAGTTGCACGGGCTTCTTGGCCT
H1-15	629:658	Upstream	CGTGAGATAAGCACATCTCACGCGCCGGGCAGTTGCA
H2-60	701:730	Downstream	ACCGGTCTTTGCAGAGACCGGTTGCCGCCGGTGGAGTGGCGGCCCTCGGCGCCGGGTTCGTACTGTTCCACGATGGTGTAGTCCT
H2-30	644:658	Downstream	ACCGGTCTTTGCAGAGACCGGTGTTCGTACTGTTCCACGATGGTGTAGTCCT
H2-15	701:715	Downstream	ACCGGTCTTTGCAGAGACCGGTCGATGGTGTAGTCCT
Helper variant 1			
H1-60	505:534 + 535:564	Upstream	GTCCCACCAAAACAGGTGGGACACACTCGCCCTTCAGCGCGCCGTCCTCGGGGTAGGGCATCCGCTCGGAGGAGGCCTCCCAGCCCAT
H2-60	606:635 + 636:665	Downstream	GACCGTCCGGGCTAGGACGGTCATTTTGTACGCGCCGGGCAGTTGCACGGGCTTCGGGTTGGCCTTGTAGGTGGTCTTGACCTCAGCG
H2-30	606:635	Downstream	GACCGTCCGGGCTAGGACGGTCATTTTGGCCTTGTAGGTGGTCTTGACCTCAGCG
H2-15	606:620	Downstream	GACCGTCCGGGCTAGGACGGTCATTGTCTTGACCTCAGCG

Supplementary Table S5. *clb* mRNA sequences used in this study. Plasmid sequences of *clb* mRNA can be constructed by replacing Table S2. with the *clb* mRNA sequences described underneath.

Name	Sequence
clbA	ATGAGGATTGATATATTAATTGGACATACTAGTTTTTTTCATCAAACCAGTAGAGATAACTTCCTT<br>CACTATCTCAATGAGGAAGAAATAAAACGCTATGATCAGTTTCATTTTGTGAGTGATAAAGAACT<br>CTATATTTTAAGCCGTATCCTGCTCAAAACAGCACTAAAAAGATATCAACCTGATGTCTCATTAC<br>AATCATGGCAATTTAGTACGTGCAAATATGGCAAACCATTTATAGTTTTTCCTCAGTTGGCAAAA<br>AAGATTTTTTTAACCTTTCCCATACTATAGATACAGTAGCCGTTGCTATTAGTTCTCACTGCGAG<br>CTTGGTGTCGATATTGAACAAATAAGAGATTTAGACAACTCTTATCTGAATATCAGTCAGCATTTT<br>TTTACTCCACAGGAAGCTACTAACATAGTTTCACTTCCTCGTTATGAAGGTCAATTACTTTTTTGG<br>AAAATGTGGACGCTCAAvAGAAGCTTACATCAAATATCGAGGTAAAGGCCTATCTTTAGGACTG<br>GATTGTATTGAATTTCATTTAACAAATAAAAAACTAACTTCAAAATATAGAGGTTCACCTGTTTAT<br>TTCTCTCAATGGAAAATATGTAACTCATTTCTCGCATTAGCCTCTCCACTCATCACCCCTAAAATA<br>ACTATTGAGCTATTTCCTATGCAGTCCCAACTTTATCACCACGACTATCAGCTAATTCATTCGTCA<br>AATGGGCAGAATTGA
clbE	ATGAAAAAGCAAGATATGAAAGCCGCCATTCGGGAATTTCTTTCACGCTCATTACGTGGGCATAC<br>GTTGAACGATGATGACGATATTTTTCTCTCGGGCTTGTCCATTCGTTATTTACTGTGCAAATCATA<br>CTGTTTATAGAAAAAAATTTTCAGGTTGAGCTGGAAGTGAGTGAGTTGAAAACAGAACAGATTG<br>CTACCGTCAATAAAATAGTGGAGCTCATTCAGCGACAAACAGGCCTGGAGTAA
clbP	ATGACAATAATGGAACACGTTAGCATTAAAACATTATATCATCTCCTGTGCTGTATGCTGCTCTTT<br>ATTTCCGCTATGTGCGCTTTGGCGCAAGAACATGAGCCTATCGGGGCGCAAGATGAGCGCCTGTC<br>GACATTAATTCACCAACGGATGCAGGAGGCCAAGGTCCCAGCCCTTTCCGTAAGTGTGACCATT<br>AAGGGGGTACGTCAGCGATTTGTCTACGGTGTTGCCGATGTGGCTAGTCAGAAAGCGAATACTCT<br>AGACACAGTTTACGAGCTGGGATCGATGAGTAAGGCGTTTACCGGACTTGTGGTGCAAATACTG<br>ATTCAGGAAGGCAGACTCCGGCAAGGGGATGATATCATTACCTATCTGCCGGAAATGCGCTTGA<br>ATTATCAGGGAAAACCTGCTTCCCTGACCGTGGCTGATTTCCTTTATCATACATCAGGATTGCCTT<br>TTTCAACACTGGCTCGGCTGGAAAACCCTATGCCTGGGAGCGCTGTGGCACAGCAACTGCGCAA<br>CGAGAATCTGCTGTTTGCGCCGGGTGCGAAGTTTAGCTATGCCTCCGCCAATTATGATGTGTTGGG<br>CGCGGTGATTGAAAATGTGACGGGAAAAACCTTTACAGAGGTCATTGCGGAACGACTCACGCAG<br>CCGCTGGGCATGTCGGCGACTGTGGCAGTTAAGGGGGATGAGATTATTGTCAACAAGGCAAGCG<br>GCTATAAACTGGGATTCGGCAAACCCGTTCTGTTTCATGCGCCTCTGGCCCGGAACCATGTTCCTG<br>CCGCCTATATCCATAGCACTCTGCCTGATATGGAAATATGGATAGACGCCTGGTTGCACAGAAAG<br>GCTTTGCCGGCAACGCTGCGTGAGGCGATGAGTAACAGTTGGCGTGGTAATAGTGATGTTCCGCT<br>TGCCGCAGACAATCGTATCCTCTATGCCAGCGGTTGGTTTATCGACCAGAATCAAGGCCCTTACA<br>TCAGTCACGGTGGGCAGAATCCAAACTTTTCTTCTTGCATTGCGTTGCGACCGGATCAGCAGATT<br>GGCATTGTTGCGCTGGCAAATATGAATTCGAATCTGATACTACAGCTTTGCGCGGATATCGATAA<br>TTATCTGCGCATTGGCAAATATGCTGACGGCGCTGGTGATGCAATTACAGCCACCGATACCCTTT<br>TCGTCTACCTCACGTTGTTGCTGTGTTTTTGGGGGGCGGTGGTTGTAGTGCGCGGTGCTTTCCGTGT<br>TTATCGCGCAACGGCGCATGGCCCTGGAAAACAGCAGAGGTTACGTTTACGCGTACGTGACTAT<br>ATCATCGCCTTGGCGGTTCCTGGGCTCGTGGCCGCCATGCTCTATGTCGCACCGGGTATACTATCT<br>CCAGGACTTGACTGGCGTTTTATCTTGGTATGGGGTCCATCGAGCGTGTTGGCGATACCGTTCGGA<br>ATTATCCTGTTAGCTTTCGTTCTGACATTAAATCATCAAATTAAACGAATTCTATTACACAACAAG<br>GAGTGGGACGATGAGTAA
clbQ	ATGAGTAATATCAGTTTGTATTGTTTGCCATATTCAGGTGGTTCTGCCGCCATGTATTATAAATGG<br>CGTAGCGTGCTGTCGGACAATATTACTTTGCGGCCTTTAGAACCTGCGGGGAGGGGAACTAGAAT<br>ACGCCAGCCGCTGTGTCTTACGATGGTGGATGCCGTCGCTGACCTTTATCAACAATTTGTGAAAC<br>ACTACACAGGTGGAGACTACGCCATTTTTGGGCATAGTCTCGGAGGGATCATGGCCTTCGAACTG<br>GTGCATTATATTCTCGATCATGGACATGACATGCCATGCGCGCTGTTTTTTTCCGGCTGTCGCCCA<br>CCCGATCGGGCCTCTCATGAAGTAATACTGCATACCTTGCCCGATCAGGCGTTTATGGAAGAGAT<br>CGTCAAGCTGGGCGGAACTCCGGTTGATGTCTTTCGTAATAAAGAGTTAATGACAATTTTCACCC

	CCATCATTAAAAACGATTATCGGCTCTATGAGCAGTATGTATTTCAGGCCAAGGCGCGCACATTA ACCTGTCCGATCGTGCTATTTCATGGCGATGCTGACAATCTGGTAATGCAGGATGAATTACTTGC ATGGGAAAAATTCACCACACGAAAGACGCGGACTATTATATTTCCTGCTGCCGATCATTTTTTTGT CGATAAGCATTTTGAACAGGTGGTAGGTTATGTGAACCAGACGATTGAATCACTCGAAATAGTA GGGTAG

Supplementary Table S6. *clb* synthetic short trigger sequences used in this study.

Name	Target site	Classification	Sequence
clbA	390:419	open	TTTTTTTACTCCACAGGAAGCTACTAACAT
	477:506	open	AGAAGCTTACATCAAATATCGAGGTAAAGG
	447:476	structured	ATTACTTTTTTGGAAAATGTGGACGCTCAA
	513:542	structured	TTTAGGACTGGATTGTATTGAATTTCATTT
	630:659	intermediate	CTCTCCACTCATCACCCCTAAAATAACTAT
clbE	1:30	open	ATGAAAAAGCAAGATATGAAAGCCGCCATT
	219:248	open	GCTCATTCAGCGACAAACAGGCCTGGAGTA
	95:124	structured	TCGGGCTTGTCCATTCGTTATTTACTGTGC
	40:69	structured	CTTTCACGCTCATTACGTGGGCATACGTTG
	83:112	intermediate	ATATTTTTTCTCTCGGGCTTGTCCATTCGT
clbP	969:998	open	TTACATCAGTCACGGTGGGCAGAATCCAAA
	992:1021	open	ATCCAAACTTTTCTTCTTGCATTGCGTTGC
	1205:1234	structured	CGGTGGTTGTAGTGCGCGGTGCTTTCCGTG
	846:875	structured	TTTGCCGGCAACGCTGCGTGAGGCGATGAG
	160:189	intermediate	GCCAAGGTCCCAGCCCTTTCCGTAAGTGTG
clbQ	256:285	open	GAACTGGTGCATTATATTCTCGATCATGGA
	506:535	open	CCAAGGCGCGCACATTAACCTGTCCGATCG
	133:162	structured	CGCCAGCCGCTGTGTCTTACGATGGTGGAT
	475:504	structured	TATCGGCTCTATGAGCAGTATGTATTTCAG
	86:115	intermediate	ATATTACTTTGCGGCCTTTAGAACCTGCGG

Supplementary Table S7. *clb* mRNA sensor sequences used in this study. Common sequence element from high-performance first-generation toehold switch was colored in yellow [3]. Conserved linker sequence was colored as gray. Trigger binding region was covered in green and its complementary region was colored in cyan. Plasmid sequences of *clb* sensor can be constructed by replacing the sensor RNA region in the example plasmids in Table S2 with the *clb* sensor sequences described underneath.

Name	Target site	Classification	Sequence
clbA	390:419	open	ATGTTAGTAGCTTCCTGTGGAGTAAAAAAAGTTATAGTTATGAGACAA GAACAGAGGAGACATAACATGAACTTTTTTAACAAAGTTAACCTGGC GGCAGCGCAAAAG
	477:506	open	CCTTTACCTCGATATTTGATGTAAGCTTCTGTTATAGTTATGAGACAAG AACAGAGGAGACATAACATGAACAGAAGCAACTCTGTTAACCTGGCG GCAGCGCAAAAG
	447:476	structured	TTGAGCGTCCACATTTTCCAAAAAAGTAATGTTATAGTTATGAGACAA GAACAGAGGAGACATAACATGAACATTACTAACAATGTTAACCTGGC GGCAGCGCAAAAG
	513:542	structured	AAATGAAATTCAATACAATCCAGTCCTAAAGTTATAGTTATGAGACA AGAACAGAGGAGACATAACATGAACTTTAGGAACAAAGTTAACCTG GCGGCAGCGCAAAAG

	630:659	intermediate	ATAGTTATTTTAGGGGTGATGAGTGGAGAGGTTATAGTTATGAGACAAGAACAGAGGAGACATAACATGAACCTCTCCAACGAGGTTAACCTGGCGGCAGCGCAAAAG
clbE	1:30	open	AATGGCGGCTTTCATATCTTGCTTTTTCATGTTATAGTTATGAGACAAGAACAGAGGAGACATAACATGAACATGAAAAACCATGTTAACCTGGCGGCAGCGCAAAAG
	219:248	open	TACTCCAGGCCTGTTTGTCGCTGAATGAGCGTTATAGTTATGAGACAAGAACAGAGGAGACATAACATGAACGCTCATAACAGCGTTAACCTGGCGGCAGCGCAAAAG
	95:124	structured	GCACAGTAAATAACGAATGGACAAGCCCGAGTTATAGTTATGAGACAAGAACAGAGGAGACATAACATGAACTCGGGCAACCGAGTTAACCTGGCGGCAGCGCAAAAG
	40:69	structured	CAACGTATGCCCACGTAATGAGCGTGAAAGGTTATAGTTATGAGACAAGAACAGAGGAGACATAACATGAACCTTTCAAACAAGGTTAACCTGGCGGCAGCGCAAAAG
	83:112	intermediate	ACGAATGGACAAGCCCGAGAGAAAAAATATGTTATAGTTATGAGACAAGAACAGAGGAGACATAACATGAACATATTTAACTATGTTAACCTGGCGGCAGCGCAAAAG
clbP	969:998	open	TTTGGATTCTGCCCACCGTGACTGATGTAAGTTATAGTTATGAGACAAGAACAGAGGAGACATAACATGAACTTACATAACCTGGCGGCAGCGCAAAAG
	992:1021	open	GCAACGCAATGCAAGAAGAAAAGTTTGGATGTTATAGTTATGAGACAAGAACAGAGGAGACATAACATGAACATCCAAAACGATGTTAACCTGGCGGCAGCGCAAAAG
	1205:1234	structured	CACGGAAAGCACCGCGCACTACAACCACCGGTTATAGTTATGAGACAAGAACAGAGGAGACATAACATGAACCGGTGGAACCCGGTTAACCTGGCGGCAGCGCAAAAG
	846:875	structured	CTCATCGCCTCACGCAGCGTTGCCGGCAAAGTTATAGTTATGAGACAAGAACAGAGGAGACATAACATGAACTTTGCCAACAAAGTTAACCTGGCGGCAGCGCAAAAG
	160:189	intermediate	CACACTTACGGAAAGGGCTGGGACCTTGGCGTTATAGTTATGAGACAAGAACAGAGGAGACATAACATGAACGCCAAGAACGGCGTTAACCTGGCGGCAGCGCAAAAG
clbQ	256:285	open	TCCATGATCGAGAATATAATGCACCAGTTCGTTATAGTTATGAGACAAGAACAGAGGAGACATAACATGAACGAACTGAACTTCGTTAACCTGGCGGCAGCGCAAAAG
	506:535	open	CGATCGGACAGGTTAATGTGCGCGCCTTGGGTTATAGTTATGAGACAAGAACAGAGGAGACATAACATGAACCCAAGGAACTGGGTTAACCTGGCGGCAGCGCAAAAG
	133:162	structured	ATCCACCATCGTAAGACACAGCGGCTGGCGGTTATAGTTATGAGACAAGAACAGAGGAGACATAACATGAACCGCCAGAACGCGGTTAACCTGGCGGCAGCGCAAAAG
	475:504	structured	ATCCACCATCGTAAGACACAGCGGCTGGCGGTTATAGTTATGAGACAAGAACAGAGGAGACATAACATGAACCGCCAGAACGCGGTTAACCTGGCGGCAGCGCAAAAG
	86:115	intermediate	CCGCAGGTTCTAAAGGCCGCAAAGTAATATGTTATAGTTATGAGACAAGAACAGAGGAGACATAACATGAACATATTAAACTATGTTAACCTGGCGGCAGCGCAAAAG

Supplementary Table S8. Helper sequences for *clb* mRNA sensor used in this study. Plasmid sequences of *clb* helper can be constructed by replacing the Helper 1 and Helper 2 region in the example plasmids in Table S2 with the appropriate helper sequences described underneath. 5′ structure was colored in green. mRNA binding regions were covered in yellow.

Name	Target site	Position	Sequence
Open			
*clbA*1	357:386	upstream	GCTCCAGCCCGCTCGCTGGAGCACCTGACTGATATTCAGATAAGAGTTGTCTAAAACC
	423:452	downstream	GGCTGCTCGGGGAGGAGCAGCCGAAAGTAATTGACCTTCATAACGAGGAAGTGAAACC
*clbA*2	444:473	upstream	GCACCGCCAAGTACGGCGGTGCCCCAGCGTCCACATTTTCCAAAAAAGTAATTGAATT
	510:539	downstream	GCCCCTACAAGTCCGTAGGGGCCTCTGAAATTCAATACAATCCAGTCCTAAAGATCCC
*clbP*1	936:965	upstream	GGTCGAGGAAAACGCCTCGACCGTACCTTGATTCTGGTCGATAAACCAACCGCTGCGC
	1002:1031	downstream	GCCCCCGCACATCAGCGGGGGCAAATGATCCGGTCGCAACGCAATGCAAGAAGAAAAG
*clbP*2	959:988	upstream	GGAGCTGCAACGGAGCAGCTCCACCGCCCACCGTGACTGATGTAAGGGCCTTGATCTA
	1025:1054	downstream	GCCGCGTCGACTAAGACGCGGCACGCCAGCGCAACAATGCCAATCTGCTGATCCGACA
*clbQ*1	223:252	upstream	GCGGTCGCTCCGCCGCGACCGCAACGGCCATGATCCCTCCGAGACTATGCCCAAAGTC
	289:318	downstream	CGCCTCGCAAAAAAGCGAGGCGTAAGCCGGAAAAAAACAGCGCGCATGGCATGTCGAT
*clbQ*2	473:502	upstream	GGACCCTCTTTGCTGAGGGTCCACCGAAATACATACTGCTCATAGAGCCGATAATACC
	539:568	downstream	GCCCAAGCCCACCCGCTTGGGCATATTACCAGATTGTCAGCATCGCCATGAAATACCC
Intermediate			
clbA	597:626	upstream	GGACGTACCATTCAGTACGTCCTTAAATGCGAGAAATGAGTTACATATTTTCCATCGG
	663:692	downstream	GACGGTCCGGCGGAGGACCGTCACATGATAAAGTTGGGACTGCATAGGAAATAGCGAA
clbE	127:156	upstream	GGGCGGCCTAACCCGGCCGCCCTTTCATCATCGTTCAACGTATGCCCACGTAATGGCT
	193:222	downstream	GCGCCCTGGCGCGACAGGGCGCACCTTTCTATAAACAGTATGATTTGCACAGTAACCC
clbP	50:79	upstream	GGGTGAGGGCGAAACCTCACCCAACCTGCATCCGTTGGTGAATTAATGTCGACAGTCC
	116:145	downstream	CAGTAGGCGACCCAGCCTACTGAGCGTAGACAAATCGCTGACGTACCCCCTTAATTAA
clbQ	53:82	upstream	GGCGGCGCGATGGAGCGCCGCCAAACCGACAGCACGCTACGCCATTTATAATACACAC
	119:148	downstream	GCCGGAGCATATCCGCTCCGGCTTCGACACAGCGGCTGGCGTATTCTAGTTCCCCCCA

Supplementary Table S9. Other accessory sequences used in this study. Accessory sequences used for constructing plasmids are indicated here.

Name	**Sequence**
RBS	AGAAGGAGA or AGAGGAGA
Linker	AACCTGGCGGCAGCGCAAAAG
T7term	TAGCATAACCCCTTGGGGCCTCTAAACGGGTCTTGAGGGGTTT TTTG
GFPmut3b_ASV	ATGCGTAAAGGAGAAGAACTTTTCACTGGAGTTGTCCCAATTC TTGTTGAATTAGATGGTGATGTTAATGGGCACAAATTTTCTGTC AGTGGAGAGGGTGAAGGTGATGCAACATACGGAAAACTTACC CTTAAATTTATTTGCACTACTGGAAAACTACCTGTTCCGTGGCC AACACTTGTCACTACTTTCGGTTATGGTGTTCAATGCTTTGCGA GATACCCAGATCACATGAAACAGCATGACTTTTTCAAGAGTGC CATGCCCGAAGGTTACGTACAGGAAAGAACTATATTTTTCAAA GATGACGGGAACTACAAGACACGTGCTGAAGTCAAGTTTGAA GGTGATACCCTTGTTAATAGAATCGAGTTAAAAGGTATTGATT TTAAAGAAGATGGAAACATTCTTGGACACAAATTGGAATACA ACTATAACTCACACAATGTATACATCATGGCAGACAAACAAA AGAATGGAATCAAAGTTAACTTCAAAATTAGACACAACATTG AAGATGGAAGCGTTCAACTAGCAGACCATTATCAACAAAATA CTCCGATTGGCGATGGCCCTGTCCTTTTACCAGACAACCATTAC CTGTCCACACAATCTGCCCTTTCGAAAGATCCCAACGAAAAGA GAGACCACATGGTCCTTCTTGAGTTTGTAACCGCTGCTGGGATT ACACATGGCATGGATGAACTATACAAAAGGCCTGCAGCAAAC GACGAAAACTACGCTGCATCAGTTTAATAA
mCherry	ATGGGCAGCAGCCATCATCATCATCATCACAGCAGCGGCCTG GTGCCGCGCGGCAGCCataTgCGtaAAATGGTGAGCAAGGGCGA AGAAGATAACATGGCCATCATCAAGGAGTTCATGCGCTTCAA GGTTCACATGGAGGGCTCCGTGAACGGCCACGAGTTCGAGAT CGAGGGCGAGGGCGAGGGCCGCCCCTACGAGGGCACCCAGA CCGCCAAGCTGAAGGTGACCAAGGGTGGCCCCCTGCCCTTCGC CTGGGACATCCTGTCCCCTCAGTTCATGTACGGCTCCAAGGCC TACGTGAAGCACCCCGCCGACATCCCCGACTACTTGAAGCTGT CCTTCCCCGAGGGCTTCAAGTGGGAGCGCGTGATGAACTTCGA GGACGGCGGCGTGGTGACCGTGACCCAGGACTCCTCCCTGCA AGACGGCGAGTTCATCTACAAGGTGAAGCTGCGCGGCACCAA CTTCCCCTCCGACGGCCCCGTAATGCAGAAGAAGACTATGGGC TGGGAGGCCTCCTCCGAGCGGATGTACCCCGAGGACGGCGCG CTGAAGGGCGAGATCAAGCAGAGGCTGAAGCTGAAGGACGG CGGCCACTACGACGCTGAGGTCAAGACCACCTACAAGGCCAA GAAGCCCGTGCAACTGCCCGGCGCGTACAACGTCAACATCAA GTTGGACATCACCTCCCACAACGAGGACTACACCATCGTGGA ACAGTACGAACGCGCCGAGGGCCGCCACTCCACCGGCGGCAT GGACGAGCTGTACAAGTAA

Supplementary Table S10. Primers used for qPCR.

Primer Name	Sequence (5′ to 3′)
*clbA*_fwd	ATTAGTTCTCACTGCGAGC
*clbA*_rev	TCTTTGAGCGTCCACATTT
*clbP*_fwd	GAGTGCTATGGATATAGGCG
*clbP*_rev	CCAATTATGATGTGTTGGGC
*clbE*_fwd	GTTTTCAACTCACTCACTTCC
*clbE*_rev	ATTTCTTTCACGCTCATTACG
*clbQ*_fwd	GGTGAATTTTTCCCATGCAA
*clbQ*_rev	AGGCGTTTATGGAAGAGATC
*mCherry*_fwd	GAAGATAACATGGCCATCATCAAGG
*mCherry*_rev	TACATGAACTGAGGGGACAGGAT
16S_rRNA_fwd	TGGTAGTCCACGCCGTAAAC
16S_rRNA_rev	TTTAACCTTGCGGCCGTACT
*cysG*_fwd	ATGCGGTGAACTGTGGAATAAACG
*cysG*_rev	CTCTTGCCATCGGATGTGCCCA

Supplementary Table S11. Gibbs free energy of mRNAs, mRNA sensors, and RNA complexes studied in this paper (unit: kcal/mol). For this calculation, full length mRNAs were used. ※ ΔΔΔG = ΔΔG_helper – ΔΔG_original.

1) [mRNA] + [toehold sensor] → [mRNA + toehold sensor].

**(kcal/mol)**	*mCherry* sensor C	A_open1	A_open2	P_open1	P_open2	Q_open1	Q_open2
**ΔG[mRNA]**	-290.431	-131.42	-131.42	-489.438	-489.438	-202.712	-202.712
ΔG[toehold sensor]	-28.137	-19.704	-26.481	-24.346	-24.507	-22.88	-37.196
ΔG[mRNA + toehold sensor]	-371.688	-187.65	-192.459	-551.231	-546.148	-267.209	-275.103
ΔΔG_original	**-53.12**	**-36.526**	**-34.558**	**-37.447**	**-32.203**	**-41.617**	**-35.195**

2) [mRNA + h1 + h2] + [toehold sensor] → [mRNA + h1 + h2 + toehold sensor]

(kcal/mol)	*mCherry* sensor C			A_open1	A_open2	P_open1	P_open2	Q_open1	Q_open2
	Helper_60	Helper_30	Helper_15						
ΔG[mRNA+ h1 + h2]	-542.652	-428.143	-367.885	-256.599	-249.457	-630.618	-636.051	-342.383	-331.403
ΔG[toehold sensor]		-28.137		-19.704	-26.481	-24.346	-24.507	-22.88	-37.196
ΔG[mRNA+h1 +h2+toehold sensor]	-627.23	-512.721	-452.463	-319.381	-313.799	-699.381	-700.853	-411.982	-409.83
ΔΔG_helper		**-56.441**		**-43.078**	**-37.861**	**-44.417**	**-40.295**	**-46.719**	**-41.231**
ΔΔΔG		**-3.321**		**-6.552**	**-3.303**	**-6.97**	**-8.092**	**-5.102**	**-6.036**

Supplementary Table S12. Performance information for a selection of toehold switches, toehold repressors, 3WJ repressors, and *clb* sensors.

Name	Type	Fold change
Toehold switch		
First-generation Toehold switch #1	Short trigger	292.0 ± 19.5
First-generation Toehold switch #2	Short trigger	279.6 ± 17.6
First-generation Toehold switch #3	Short trigger	265.3 ± 28.2
Second-generation Toehold switch #1	Short trigger	665 ± 135
Second-generation Toehold switch #2	Short trigger	586 ± 92
Second-generation Toehold switch #3	Short trigger	557 ± 68
mRNA sensor derived from toehold switch		
mCherry mRNA sensor #1	Long trigger	57 ± 8
mCherry mRNA sensor #2	Long trigger	38 ± 2
mCherry mRNA sensor #3	Long trigger	17 ± 1
cat mRNA sensor	Long trigger	7 ± 3
aadA mRNA sensor	Long trigger	6 ± 1
Toehold repressor		
Toehold repressor #1	Short trigger	172.8 ± 29.4
Toehold repressor #2	Short trigger	93.3 ± 26.6
Toehold repressor #3	Short trigger	88.5 ± 17.4
mRNA sensor derived from toehold repressor		
kanR mRNA sensor	Long trigger	14 ± 2
bla mRNA sensor	Long trigger	6 ± 3
3WJ repressor		
3WJ repressor #1	Short trigger	134.2 ± 8.1
3WJ repressor #2	Short trigger	85.2 ± 4.9
3WJ repressor #3	Short trigger	66.1 ± 3.2
mRNA sensor derived from 3WJ repressor		
kanR mRNA sensor	Long trigger	20 ± 4
aadA mRNA sensor	Long trigger	7 ± 1
Switch used in this paper		
clbP sensor open #1	Short trigger	942.5 ± 99.9
clbP sensor open #2	Short trigger	75.9 ± 11.2
clbQ sensor open #1	Short trigger	259.2 ± 22.3
Switch with full length mRNA without helper		
clbP sensor open #1 without helper	Long trigger	35.8 ± 9.8
clbP sensor open # without helper	Long trigger	4.7 ± 0.8
clbQ sensor open #1 without helper	Long trigger	11.3 ± 1.1
Switch with full length mRNA with helper		
clbP sensor open #1 with helper	Long trigger	62.3 ± 16.8
clbP sensor open #2 with helper	Long trigger	57.0 ± 9.2
clbQ sensor open #1 with helper	Long trigger	15.8 ± 1.5

Supplementary Figures

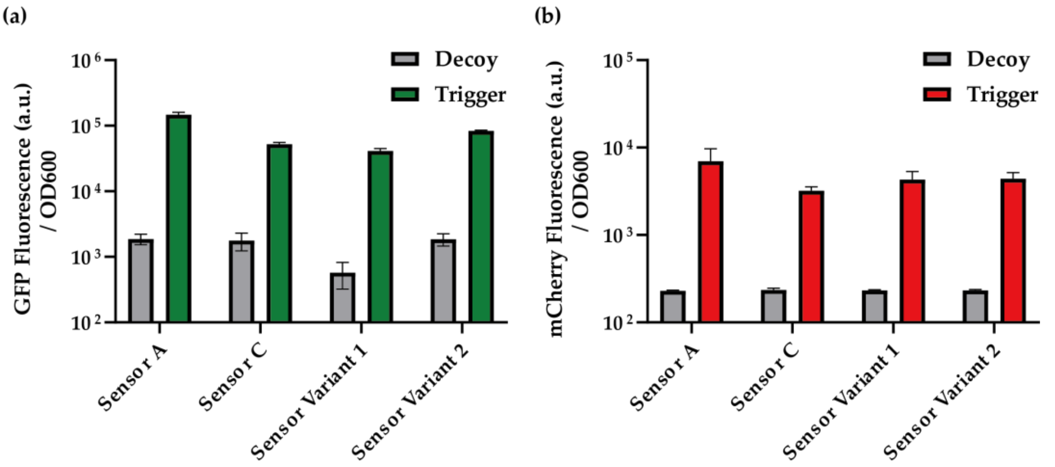

Supplementary Figure S1. *mCherry* mRNA sensor variants tested in this study. Sensor A and Sensor C were adopted from the previous research [5]. Among four sensors, sensor C and sensor variant 1 were selected for further helper tests. 0.2% (*w*/*v*) Arabinose was treated for induction of T7 RNA polymerase in *E. coli* BL21 AI strain. GFP and mCherry fluorescence were measured on the microplate reader (error bars indicate ±SD).

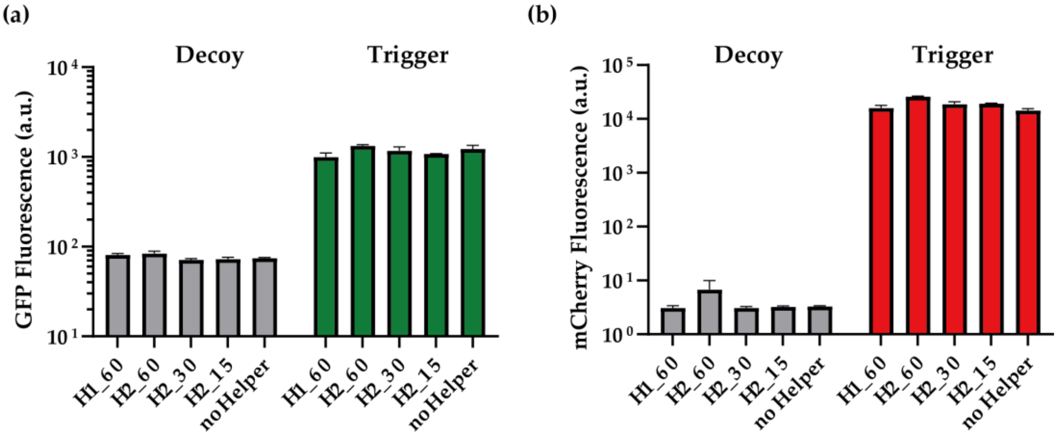

Supplementary Figure S2. Performance of *mCherry* sensor variant 1 with helper RNAs. H1 and H2 mean a helper RNA strand that stretching the upstream or downstream of a target site, respectively, and the following number means the length of the helper, 60, 30, and 15 nt. 0.2% (*w*/*v*) Arabinose was treated for induction of T7 RNA polymerases in *E. coli* BL21 AI strain. GFP and mCherry fluorescence were measured on flow cytometry (error bars indicate ±SD).

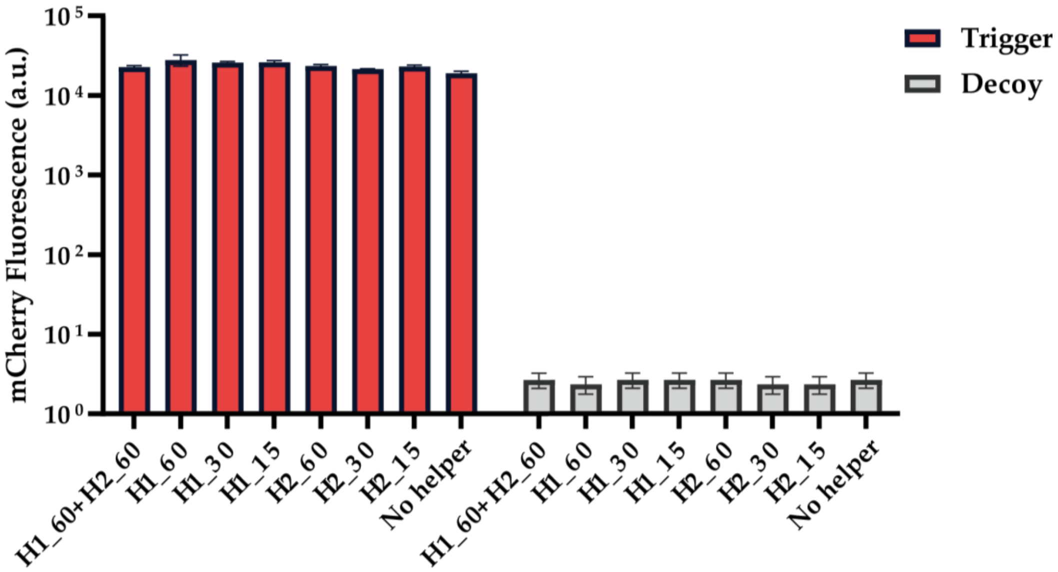

Supplementary Figure S3. mCherry expression levels in the presence of helper RNA variants. No significant inhibition of mCherry mRNA translation was observed regardless of the length or position of helper RNAs. H1 and H2 mean a helper that stretching the upstream or downstream of a toehold switch target site, respectively, and the following number means the length of the helper, 60, 30, and 15 nt. 0.2% (*w*/*v*) Arabinose was treated for induction, and GFP and mCherry fluorescence were measured on flow cytometry (error bars indicate ±SD).

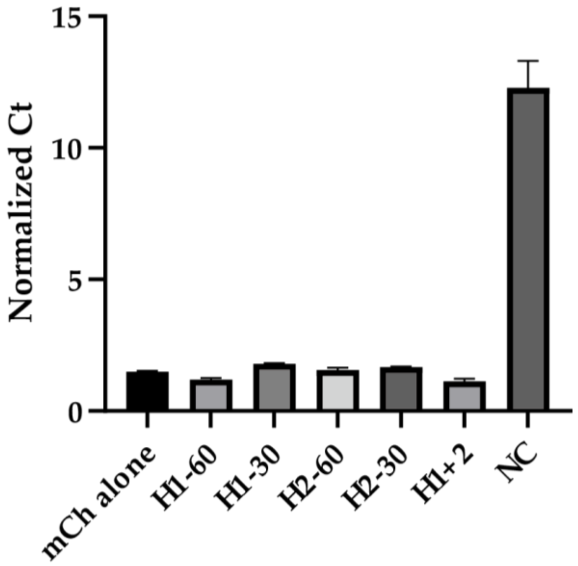

Supplementary Figure S4. RT-qPCR data for *mCherry* mRNA levels. 16s rRNA was selected as reference RNA for normalization of Ct values. *mCherry* mRNA amount was not significantly changed when it was compared to no helper (*mCherry* only) sample. Primer sequences used are listed in Table S10 (error bars indicate ±SEM).

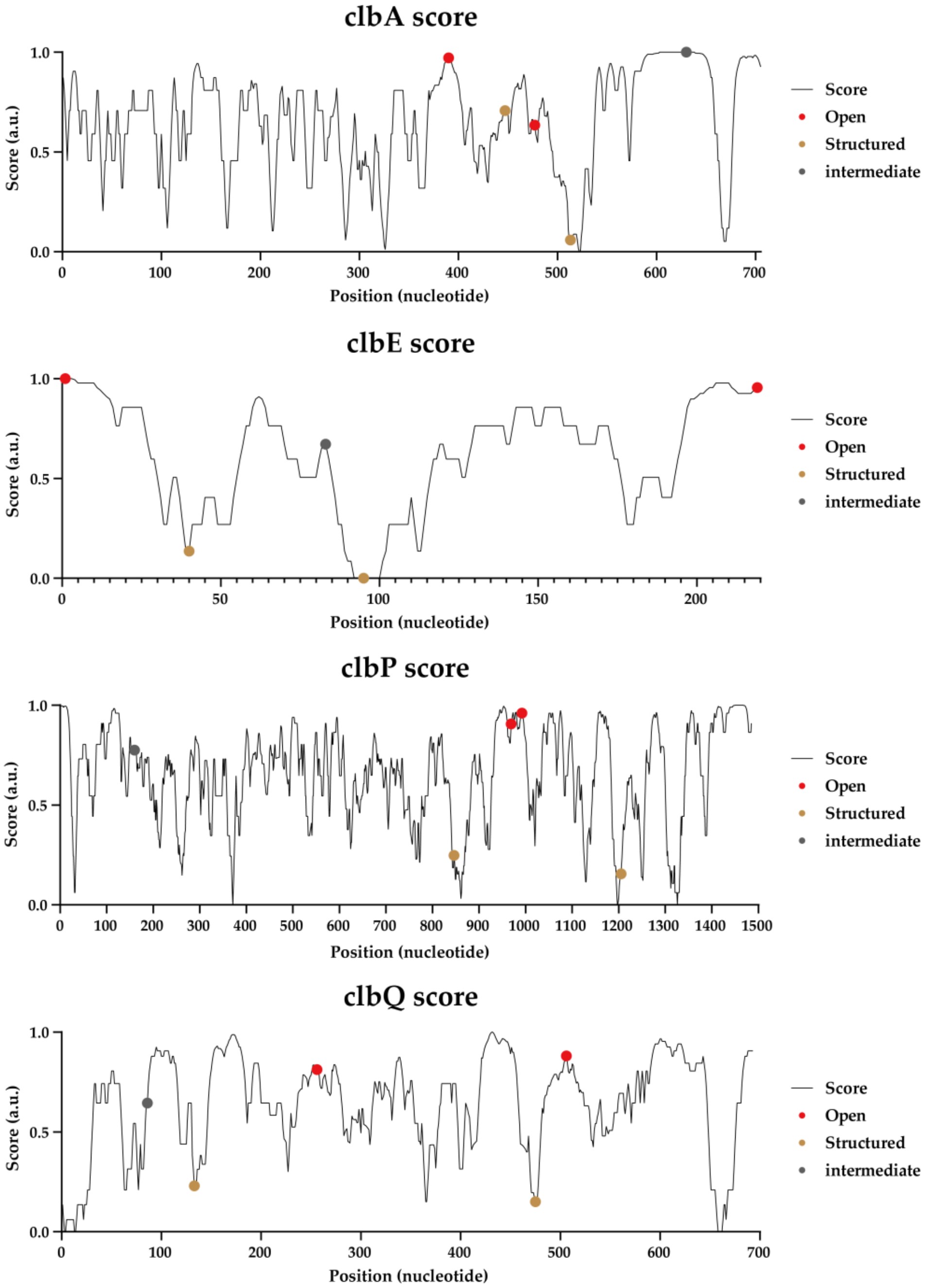

Supplementary Figure S5. Accessibility calculation of *clbA*, *clbE*, *clbP*, and *clbQ*. High score means highly single-stranded and low score means highly structured. Each point indicates the start position of the target region on a designed sensor. Color of the dot indicates the type of sensor; red: open structure, brown: highly structured, and gray: intermediate region.

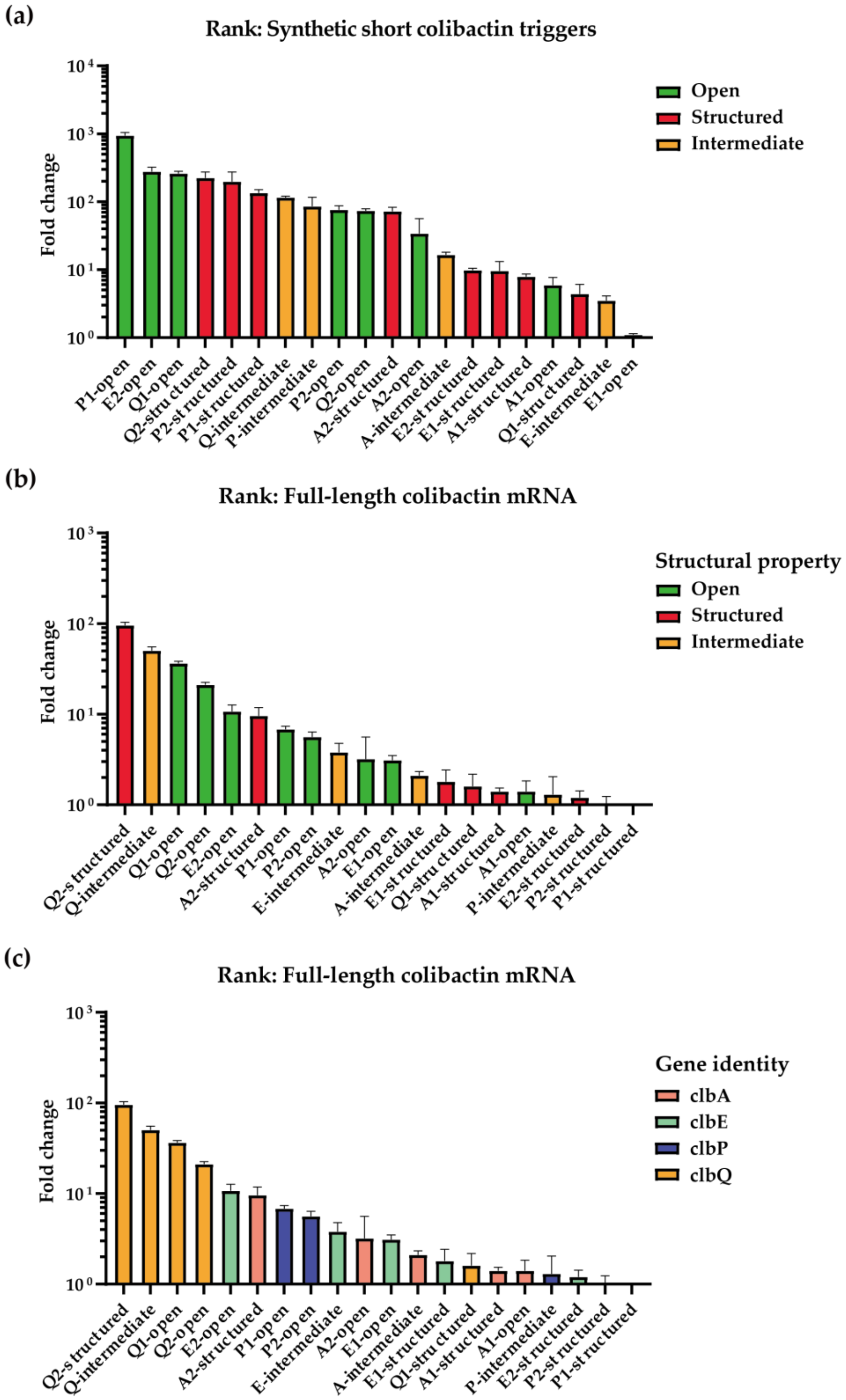

Supplementary Figure S6. In vivo performance of *clb* mRNA sensors. When cognate short triggers were treated, there were no significant difference between open region targeting sensors, close region targeting sensors and intermediated region targeting sensors was observed. However, when cognate full-length mRNAs were treated with sensors, open region targeting sensors generally showed higher performance (error bars indicate ±SEM).

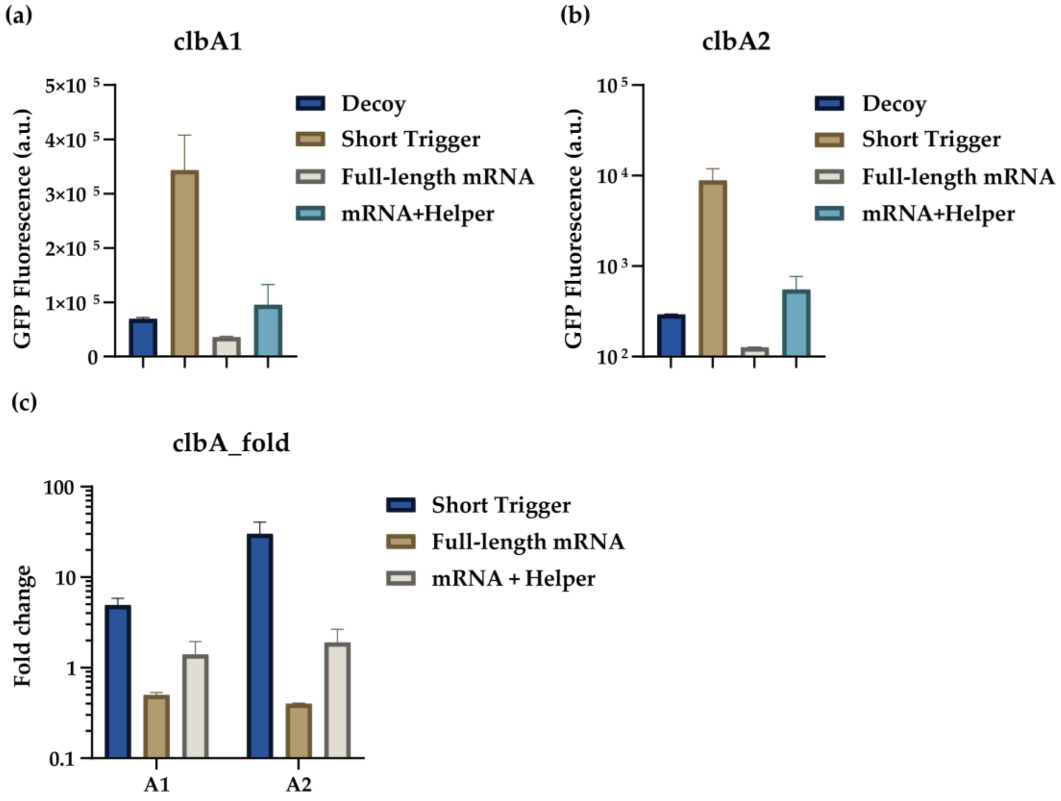

Supplementary Figure S7. In vivo performance of *clbA* open sensors with helper RNAs. Although *clbA*1 (open) and *clbA*2 (open) sensors worked well with synthetic short triggers but did not detect a full-length mRNA. Fold change was partly restored by introducing helper RNAs (error bars indicate ±SEM).

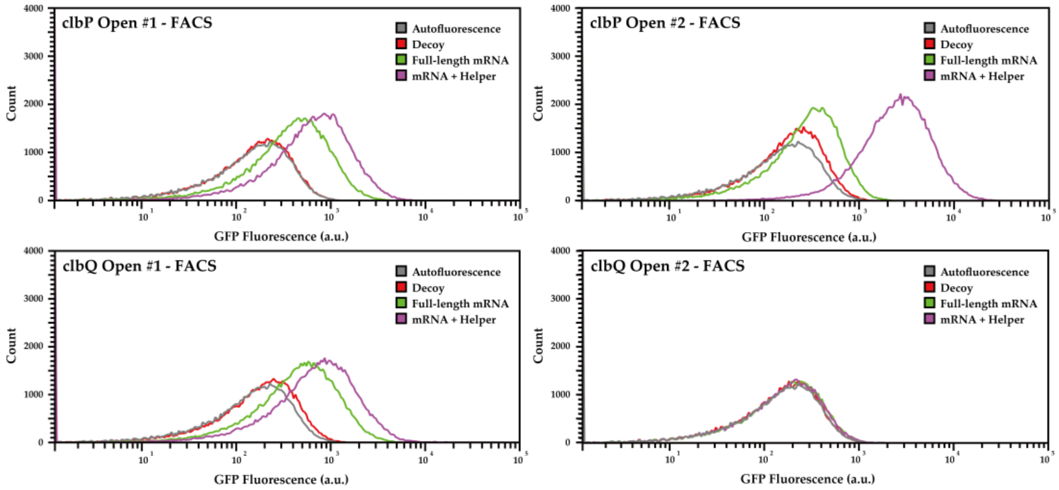

Supplementary Figure S8. Flow cytometry data for *clbP* and *clbQ* open sensors with helper RNAs. Cell population was unimodal, and GFP fluorescence increased with the introduction of helper RNAs. In all in vivo experiments, the number of biological replicates is three, and the figure showed a representative case.

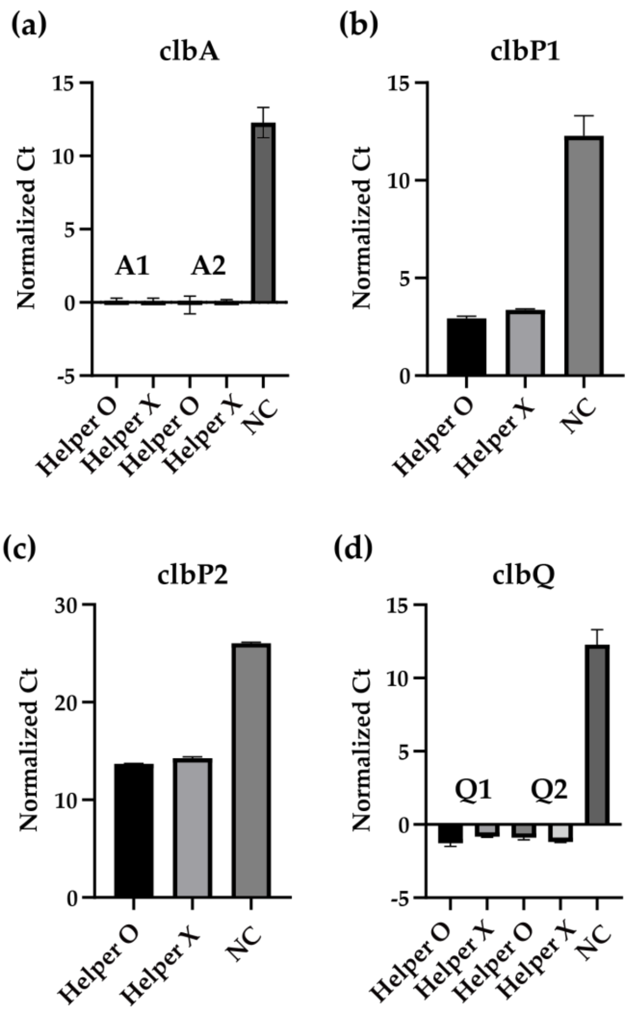

Supplementary Figure S9. RT-qPCR data for colibactin mRNA levels. 16s rRNA (for *clbA*, *clbP*1 and *clbQ*) or *CysG* (*clbP*2) were selected as reference RNA for normalization of Ct values. No significant change of target mRNA amount was observed between 'without helper' samples and 'with helper' samples. Primer sequences used are listed in Table S10 (error bars indicate ±SEM).

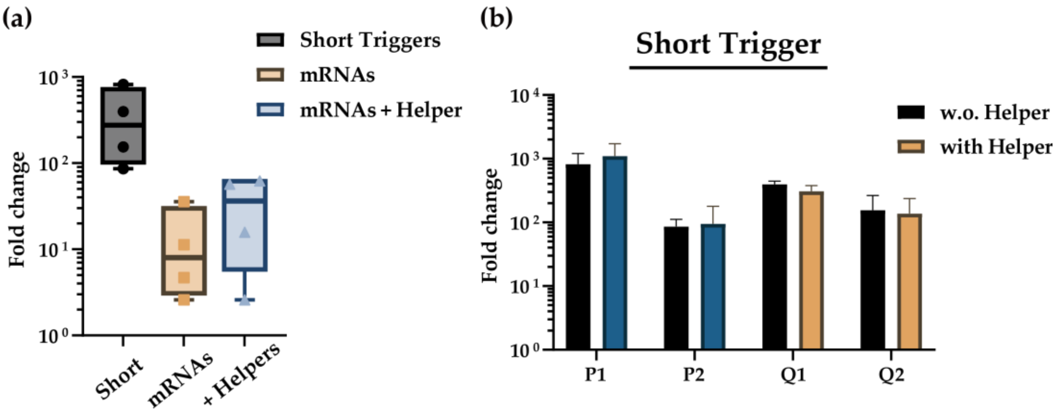

Supplementary Figure S10. In vivo performance of *clbP* and *clbQ* open sensors with short triggers and helper RNAs. (a) The sensing ability of toehold switch sensors was reduced in the full-length mRNA compared to synthetic short trigger. However, it was partially restored with the introduction of helper RNAs. (b) The sensing ability against synthetic short trigger was not affected by helper RNAs (error bars indicate ±SEM).

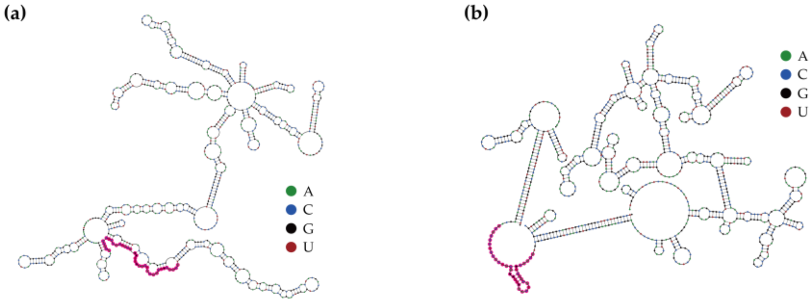

Supplementary Figure S11. Predicted MFE structures for *mCherry* mRNA without (a) and with (b) helper RNAs. Target sequences for toehold sensors are highlighted with pink. NUPACK was used to calculate MFE structures for each complex.

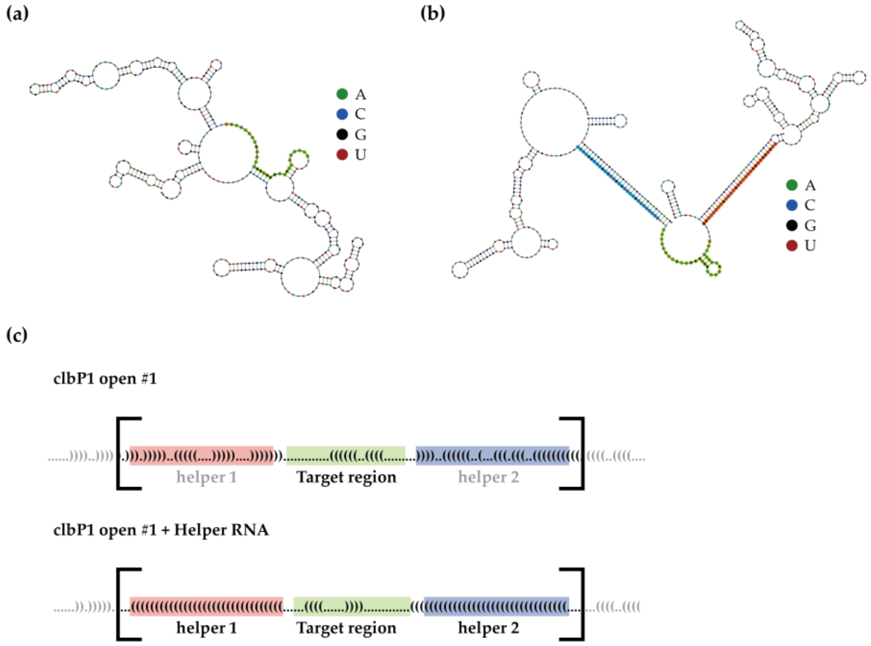

Supplementary Figure S12. Predicted MFE structure for *clb* mRNAs without and with helper RNAs. (a) *clbP* mRNA without helper RNAs for *clbP* open 1 sensor. (b) *clbP* mRNA with helper RNAs for *clbP* open1 sensor. (c) Dot-bracket model-expressed secondary structure of *clbP* open 1 sensor targeting *clbP* mRNA without or with helper RNAs. NUPACK was used to calculate MFE structures for each complex.

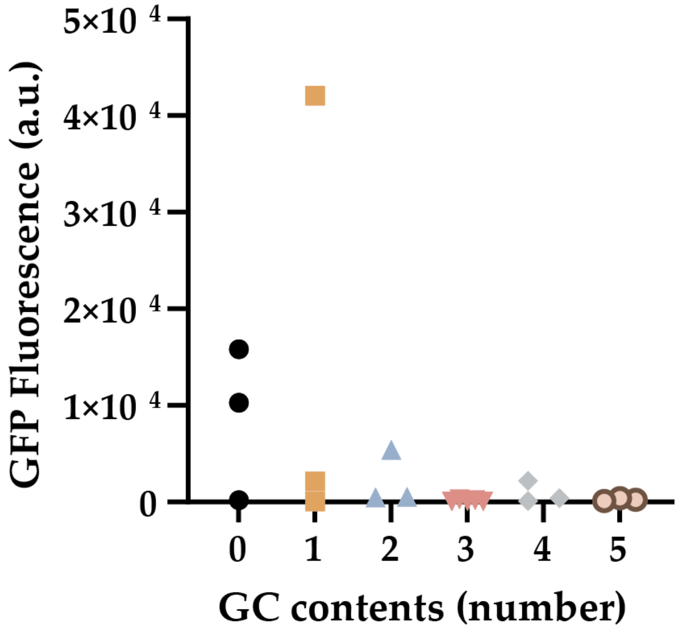

Supplementary Figure S13. The OFF-state fluorescence of *clb* sensors and the GC content of lower stem. The two cases showing highest leakages have 0 or 1 GC base-pair. The first one was *clbA* open Scheme 1. and the second one was *clbE* open sensor 1.

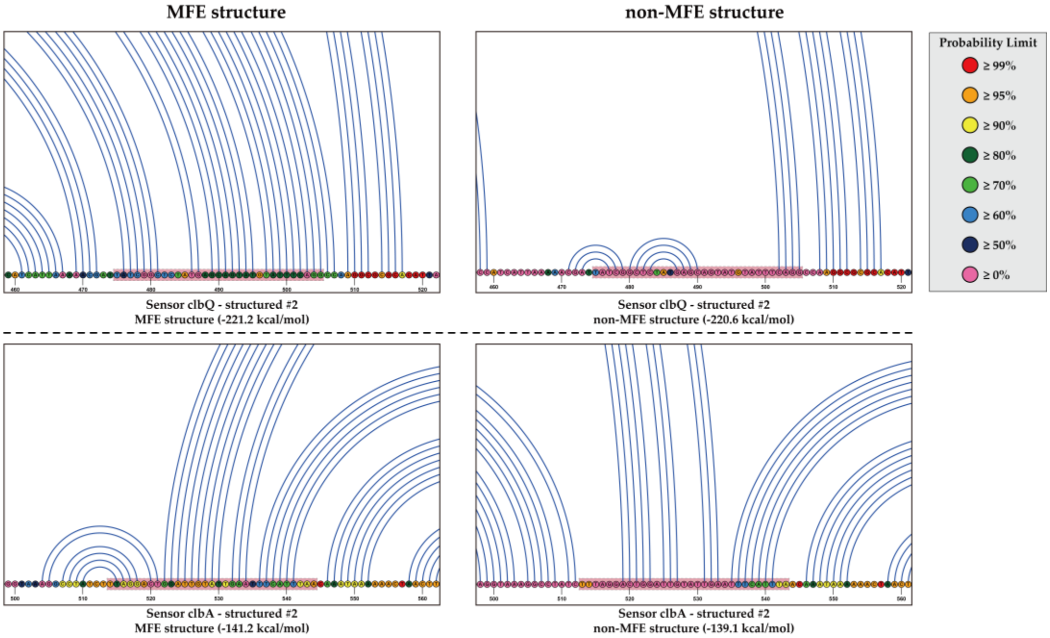

Supplementary Figure S14. Predicted alternative secondary structures for *clbQ* and *clbA* mRNAs. Secondary structure nearby target site (emphasized with red box) is dramatically changed with a small energy penalty. Structures were calculated using the RNAstructure 6. 2 [6,7] to figure out non-minimum free energy (MFE) structures.

References

1. Quan, J.; Tian, J. Circular polymerase extension cloning for high-throughput cloning of complex and combinatorial DNA libraries. *Nat Protoc* **2011**, *6*, 242–251, doi:10.1038/nprot.2010.181.
2. Gibson, D.G.; Young, L.; Chuang, R.-Y.; Venter, J.C.; Hutchison, C.A.; Smith, H.O. Enzymatic assembly of DNA molecules up to several hundred kilobases. *Nature methods* **2009**, *6*, 343–345.
3. Liu, H.; Naismith, J.H. An efficient one-step site-directed deletion, insertion, single and multiple-site plasmid mutagenesis protocol. *BMC Biotechnol* **2008**, *8*, 91, doi:10.1186/1472-6750-8-91.
4. Andersen, J.B.; Sternberg, C.; Poulsen, L.K.; Bjørn, S.P.; Givskov, M.; Molin, S. New unstable variants of green fluorescent protein for studies of transient gene expression in bacteria. *Applied and environmental microbiology* **1998**, *64*, 2240–2246.
5. Green, A.A.; Silver, P.A.; Collins, J.J.; Yin, P. Toehold switches: de-novo-designed regulators of gene expression. *Cell* **2014**, *159*, 925–939, doi:10.1016/j.cell.2014.10.002.
6. Reuter, J.S.; Mathews, D.H. RNAstructure: software for RNA secondary structure prediction and analysis. *BMC Bioinformatics* **2010**, *11*, 129, doi:10.1186/1471-2105-11-129.
7. Mathews, D.H.; Disney, M.D.; Childs, J.L.; Schroeder, S.J.; Zuker, M.; Turner, D.H. Incorporating chemical modification constraints into a dynamic programming algorithm for prediction of RNA secondary structure. *Proc Natl Acad Sci U S A* **2004**, *101*, 7287–7292, doi:10.1073/pnas.0401799101.
